# Ryanodine receptor-active non-dioxin-like polychlorinated biphenyls cause neurobehavioral deficits in larval zebrafish

**DOI:** 10.3389/ftox.2022.947795

**Published:** 2022-10-06

**Authors:** Bianca Yaghoobi, Galen W. Miller, Erika B. Holland, Xueshu Li, Danielle Harvey, Shuyang Li, Hans-Joachim Lehmler, Isaac N. Pessah, Pamela J. Lein

**Affiliations:** ^1^ Department of Molecular Biosciences, School of Veterinary Medicine, University of California, Davis, Davis, CA, United States; ^2^ Department of Biological Sciences, California State University of Long Beach, Long Beach, CA, United States; ^3^ Department of Occupational and Environmental Health, College of Public Health, The University of Iowa, Iowa City, IA, United States; ^4^ Department of Public Health Sciences, University of California, Davis, Davis, CA, United States

**Keywords:** developmental neurotoxicity, larval zebrafish, polychlorinated biphenyls, ryanodine receptor, photomotor behavior

## Abstract

Although their production was banned in the United States in 1977, polychlorinated biphenyls (PCBs) continue to pose significant risks to the developing nervous system. Perinatal exposure to PCBs is associated with increased risk of neuropsychiatric disorders, perhaps due to altered patterns of dendritic arborization of central neurons. Non-dioxin-like (NDL) PCB congeners enhance dendritic arborization of developing mammalian neurons via sensitization of ryanodine receptors (RYR). Structure-activity relationships (SAR) of RYR sensitization by PCBs have been demonstrated using mammalian and rainbow trout (*Oncorhynchus mykiss*) tissue homogenates. The purpose of this study is to determine whether this SAR translates to developmental neurotoxicity (DNT) of PCBs *in vivo*, a question that has yet to be tested. To address this gap, we leveraged a zebrafish model to evaluate the developmental neurotoxicity potential of PCBs 28, 66, 84, 95, 138, and 153, congeners previously shown to have broadly different potencies towards sensitizing RYR. We first confirmed that these PCB congeners exhibited differing potency in sensitizing RYR in zebrafish muscle ranging from negligible (PCB 66) to moderate (PCB 153) to high (PCB 95) RYR activity. Next, enzymatically dechorionated embryos were statically exposed to varying concentrations (0.1–10 μM) of each PCB congener from 6 h post-fertilization to 5 days post-fertilization (dpf). Embryos were observed daily using stereomicroscopy to assess mortality and gross malformations and photomotor behavior was assessed in larval zebrafish at 3, 4, and 5 dpf. The body burden of each PCB was measured by gas chromatography. The key findings are: 1) None of these PCBs caused death or overt teratology at the concentrations tested; 2) A subset of these PCB congeners altered photomotor behavior in larval zebrafish and the SAR for PCB behavioral effects mirrored the SAR for RYR sensitization; and 3) Quantification of PCB levels in larval zebrafish ruled out the possibility that congener-specific effects on behavior were due to differential uptake of PCB congeners. Collectively, the findings from this study provide *in vivo* evidence in support of the hypothesis that RYR sensitization contributes to the DNT of PCBs.

## Introduction

Despite being banned from production in the United States since the late 1970s and worldwide in the early 2000s through the Stockholm Convention on Persistent Organic Pollutants, polychlorinated biphenyls (PCBs) remain persistent environmental toxicants (Hu et al., 2011; Martinez et al., 2012; Koh et al., 2015; Jahnke and Hornbuckle 2019) that pose significant risk to the developing nervous system (Pessah et al., 2019; Panesar et al., 2020). Exposure to PCBs *in utero* or during infancy is associated with neurological deficits in humans (Berghuis et al., 2014; Berghuis et al., 2015; Kyriklaki et al., 2016; Lyall et al., 2017; Ikeno et al., 2018). Experimental animal studies recapitulate the neurobehavioral effects of developmental PCB exposures, and of the 209 possible congeners, non-dioxin-like (NDL) PCBs have been most strongly linked to developmental neurotoxicity (DNT) (Klocke and Lein 2020). NDL PCBs have been shown to alter neuronal connectivity and plasticity in the developing brain (Schantz et al., 1997; Schantz et al., 2003; Kenet et al., 2007; Yang et al., 2009), an effect shown *in vitro* to be mediated by sensitization of the ryanodine receptor (RYR), which enhances dendritic arborization (Wayman et al., 2012b). RYRs are integral membrane Ca^2+^ channels that directly affect the amplitude and spatiotemporal patterns of intracellular Ca^2+^ fluxes (Pessah et al., 2010). NDL PCBs bind to and sensitize RYRs, increasing their sensitivity to Ca^2+^ and stabilizing the RYR channel in its open conformation (Wong et al., 1997). PCB 95, a NDL congener with particularly potent RYR activity, enhances dendritic outgrowth of rat hippocampal and cortical neurons both *in vitro* and *in vivo* via RYR-dependent transcriptional and translational mechanisms (Wayman et al., 2012a; Wayman et al., 2012b; Keil et al., 2018).

Structure-activity relationship (SAR) studies of PCB RYR sensitization in mammalian cell culture (Wong et al., 1997; Pessah et al., 2006) demonstrated that a minimum of one *ortho* substitution along with a *meta*-substitution adjacent to the *ortho*-substitution and a lack of *para*-substitutions on the biphenyl ring increased activity at the RYR, which in turn dysregulated cellular Ca^2+^ signaling (Pessah et al., 2006; Holland et al., 2017a). However, whether this SAR translates to the *in vivo* developmental neurotoxicity of PCBs has yet to be tested. Low throughput and high costs make performing extensive *in vivo* SAR studies in rodent models challenging. In addition, strategies to assess the role of RYR in NDL PCB developmental neurotoxicity (DNT) *in vivo* using RYR mutant or knockout models or pharmacologic inhibition of RYR have not been successful because of embryonically lethality (Takeshima et al., 1994; Takeshima et al., 1998; Chelu et al., 2006) or cardiotoxicity. To address this gap, we leveraged larval zebrafish, an established *in vivo* model of DNT (Tal et al., 2020), to determine whether the SAR of PCB activity at the RYR is predictive of the *in vivo* DNT of NDL PCBs.

Zebrafish express homologs for 70% of human genes (Howe et al., 2013), and developmental signaling and stages of development are highly conserved between zebrafish, humans and other vertebrate models (Gilbert, 2010). The zebrafish has highly conserved orthologs to human RYR genes with a and b paralogs for the mammalian RYR1, RYR2, and RYR3 (Holland et al., 2017b). These paralogs exist due to whole-genome duplication events in teleost (Postlethwait et al., 2000). All RYR paralogs are expressed throughout early development (Wu et al., 2011). Some paralogs are provided maternally, but zygotic expression commences as early as 11 h post-fertilization (hpf) and as late as 18 hpf. During embryonic development, paralog expression is relatively tissue-specific, with *RYR1a* and *RYR1b* most highly expressed in slow- and fast-twitch skeletal muscle, respectively, *RYR2a* highly expressed in the brain*, RYR2b* confined to the heart, and *RYR3a* (previously described as *RYR3*) highly expressed throughout various tissues in the body (Wu et al., 2011). By adulthood, expression is more heterogeneous, and specifically, all paralogs are present in the brain (Darbandi and Franck 2009; Furlan et al., 2020).

Here, we tested the hypothesis that PCB DNT in embryonic zebrafish mirrors the SAR for PCB sensitization of RYR (Pessah et al., 2006). The congeners evaluated in this study (PCBs 28, 66, 84, 95, 138, and 153) were chosen to reflect a range of *in vitro* potency at the RYR (Pessah et al., 2006; Holland et al., 2017a), with PCBs 28 and 66 exhibiting negligible RYR activity, PCBs 138 and 153, moderate activity, and PCBs 84 and 95, potent RYR activity. These congeners have at least one chlorine in the *ortho* position, with half the congeners having adjacent *meta* substitutions (PCBs 84, 95, 138), and two lacking *para* substitutions (PCBs 84 and 95). Collectively, these congeners represent a predominant component of PCB mixtures found in environmental samples and biological tissues (Pessah et al., 2010). The direct effect of these selected PCB congeners on RYR activity in zebrafish was measured via a ryanodine binding assay in adult zebrafish muscle. Congeners were then tested for DNT using a static developmental exposure paradigm starting at 6 hpf through 5 days post-fertilization (dpf) during which teratological effects were assessed daily and photomotor behavior was assessed at 3, 4, and 5 dpf. The body burden of each congener was also assessed to confirm that the results were not confounded by differential uptake. Based on a ranking of neurotoxic potency based on our assay results, our data support the hypothesis that the SAR of PCB DNT parallels that of PCB potency at the RYR.

## Materials and methods

### Fish husbandry

Fish husbandry, spawning, and all experiments were performed in accordance with the University of California, Davis Institutional Animal Care and Use Committee (IACUC) protocols 17645 and 19391. Wildtype zebrafish (Tropical 5D strain) were originally obtained from the Sinnhuber Aquatic Research Laboratory at Oregon State University with subsequent generations raised at UC Davis. Zebrafish were maintained under standard laboratory conditions with 14 h light (∼850 lux)/10 h dark cycles (Harper and Lawrence 2011). Water was maintained at 28.5 ± 0.5°C, pH 7.2 ± 0.4 and conductivity at 700 ± 100 µS using bicarbonate and Instant Ocean Sea Salt (Blacksburg, VA, United States), respectively. Adult fish were fed twice daily with a combination of live *Artemia nauplii* (*INVE* Aquaculture, Inc., Salt Lake City, UT, United States) and commercial flake food comprised of Zeigler Zebrafish Granule (Zeigler Bros, Inc., Gardners, PA, United States), Spirulina Flake (Zeigler Bros, Inc.), Cyclopeeze (Argent Aquaculture, Redmond, WA, United States), and Golden Pearl (Brine Shrimp Direct, Ogden, UT, United States). Embryos were obtained by natural group spawning in system fish water (FW) and kept in an incubator at 28.5°C.

### PCB congeners

PCB congeners 28, 66, 95, and 153 were purchased from AccuStandard (Accustandard Inc., New Haven, CT, United States), while PCBs 84 and 138 were synthesized at the University of Iowa as described previously (Sethi et al., 2019). The purity of PCBs was determined on an Agilent 6890 chromatograph coupled with an Agilent 5975 Inert Mass Selective Detector (Agilent Technologies, Santa Clara, CA, United States) operated in electron ionization mode using a capillary Supelco SLB-5MS GC column (30 m × 250 μm × 0.25 µm, Sigma-Aldrich). Purity was determined in the total ion chromatogram (TIC) mode using the following temperature program (Telu et al., 2010; Holland et al., 2017a; Li et al., 2018): starting temperature 50°C, 10°C/min to 150°C, then 5°C/min to 280°C, hold for 6 min, and 10°C/min to 300°C, hold for 5 min. Helium flow rate was 2 ml/min. The inlet and auxiliary temperatures were both 280°C. The purity was calculated based on the relative peak area. A congener specific analysis method in the select ion monitoring (SIM) mode was employed to identify PCB impurities present in the PCB samples (Hu et al., 2015; Holland et al., 2017a). This method allowed the analysis of 209 PCB congeners as 162 peaks of individual or co-eluting peaks. The temperature program was modified based on the published method: hold at 80°C for 1 min, 2°C/min to 160°C, 1°C/min to 170°C and hold for 15 min, 1°C/min to 180°C and hold for 15 min, 1°C/min to 245°C, then 10°C/min to 300°C and hold for 15 min. The helium flow rate was 1.5 ml/min. The injector temperature was 280°C. The MS temperatures were 280°C, 230°C, and 150°C for transfer line, source and quadrupole, respectively. The GC analysis showed that all six PCB congeners had a purity >99.9%, and that no other PCB congeners were detected. The gas chromatograms and the mass spectra for all PCB congeners are shown in Supplementary Figures S1–S12. PCB congeners were dissolved in 100% DMSO (Sigma-Aldrich, St. Louis, MO, United States) and stored in amber glass vials as 1000X stocks. On exposure days, stocks were freshly diluted at 2X in embryo media (EM) (Westerfield and ZFIN, 2000).

### Ryanodine-ligand binding assay

Sensitization of the zebrafish RYR by *ortho*-PCBs was measured using [^3^H]ryanodine ([^3^H]Ry) ligand binding assays as previously described (Fritsch and Pessah, 2013). Briefly, crude microsomal protein preparations were created from the tail (post dorsal fin) muscle (primarily white skeletal muscle) of six adult zebrafish (∼1 year old) that was homogenized in a buffer containing 320 mM sucrose, 5 mM HEPES, 1 mM PMSF, 10 mM NaF, 2 mM β-Glycerol, 5 mM Na_4_P_2_O_7_, 0.5 mM Na_3_VO_4_ and 2 μg/L leupeptin (pH 7.2). The homogenate was centrifuged at 5,000 × g (4°C) for 15 min. The pellet was re-homogenized in 5 ml homogenization buffer and centrifuged at 5,000 × g. Supernatants were combined for ultracentrifugation at 100,000 g (4°C for 1 h). The resultant pellet was re-suspended in a buffer of 320 mM sucrose and 5 mM HEPES (Sigma-Aldrich, St. Louis, MO, United States) at pH 7.2. Protein concentrations were determined in triplicate using a BCA Assay (Fisher Scientific, Waltham, MA, United States). [^3^H]Ry ligand binding assays were conducted with 40 μg/ml of the crude protein homogenate in the presence of DMSO (0.5%) or 0.1–50 μM of individual PCB congeners in 0.5% DMSO. Tests were run using 5 nM [^3^H]Ry (Perkin Elmer Waltham, MA, United States), 250 mM KCl, 20 mM HEPES, 15 mM NaCl, and 50 μM CaCl_2_ (pH 7.1) and incubated for 16 h at 25°C. Non-specific binding was determined with the addition of 10 µM ryanodine (Tocris Bioscience, Bristol, United Kingdom) and 200 μM EGTA (Sigma-Aldrich, St. Louis, MO, United States). Assays were terminated by rapid filtration through Whatman GF/B filter paper (Brandel, Gaithersburg, MD, United States) and washed with ice-cold buffer containing 140 mM KCl, 10 mM HEPES, and 0.1 mM CaCl_2_, pH 7.4. Bound radioligand was measured by liquid scintillation counting (Beckman Coulter, Indianapolis, IN, United States). PCB-induced [^3^H]Ry binding was assessed in two different protein preparations, and each assay was run in triplicate. Individual PCB concentration-response curves were determined using non-linear regression (GraphPad Prism 6.0, GraphPad Software Inc., San Diego, CA, United States).

### Chemical exposures

At 4 hpf, embryos were treated with 50 µl of 41 mg/ml protease from *Streptomyces griseus* (P5147, Sigma-Aldrich, St. Louis, MO, United States) in 25 ml of FW for a maximum of 6 min to remove the chorion (Truong et al., 2011). At 5 hpf, embryos were placed in individual wells of 96-well plates filled with 100 µl of EM. At 6 hpf, embryos were exposed to a 2× concentration of the selected PCB congeners or controls that were diluted in 100 μl EM to produce a 1× concentration in a total of 200 µl solution. Exposure concentrations were 0 (DMSO control), 0.1, 0.3, 1, 3, and 10 μM. After exposure, plates were covered with Parafilm M (Bemis NA, Neenah, WI, United States) to reduce evaporation and placed in an incubator at 28.5°C with 14 h light (∼300 lux)/10 h dark cycles. Final DMSO concentrations were 0.2% (vol/vol) for all exposures. Fish were statically exposed until 5 dpf.

### Teratological and mortality assessments

Exposed fish were examined daily for any gross morphological malformations and mortality using an Olympus Stereo Microscope Model SZ61 (Olympus, Japan) up to ×4.5 magnification. On days of behavioral testing (3, 4, and 5 dpf), examination was conducted immediately after the conclusion of the behavioral assay. Teratological endpoints that were evaluated include yolk sac edema, pericardial edema, somite formation, deformities of the body axis, notochord, craniofacial structures, or caudal fin. Malformations were scored in a binary manner of either present or not present. Fish that were scored as neither malformed nor dead were deemed viable. For each exposure, the number of fish that were viable, malformed, or dead was divided by the total number of biological replicates within each group and multiplied by 100 to obtain percent incidence.

### Larval photomotor behavior testing

Larval locomotor tests were performed at 3, 4, and 5 dpf. Vehicle and PCB-exposed larvae in 96-well plates were placed in a DanioVision behavior system (Noldus Information Technology, Leesburg, VA, United States) and subjected to a 35 min light/dark locomotor test. System and plate temperature were maintained at 28.5 ± 0.5°C using a temperature control unit from Noldus. The testing paradigm consisted of a 10 min light period (∼1,900 lux) to allow for acclimation (5 min) and to record baseline swimming (5 min). This was followed by a 5 min dark period (∼0 lux) to stimulate increased swimming behavior and a 5 min light period (∼1,900 lux) to stimulate freezing behavior. The last 15 min consisted of a dark period (∼0 lux) to observe increased swimming behavior and acclimation to the dark conditions.

The distance traveled binned per minute by each larva during the 35-min observation test period was recorded via continuous tracing using a DanioVision system camera with an infrared filter to allow recording in both light and dark conditions. Movement was tracked using EthoVisionXT software (Noldus Information Technology) and exported to Excel and Prism 6.0 (GraphPad Software) for analysis and visualization. Dead and/or malformed larvae (as noted by observation under dissecting scope) were excluded from the behavior analysis.

The trapezoidal rule was used to compute the area under the curve (AUC) for each fish per day per lighting condition. The first 5 min of the 35-min observation period was removed to account for initial acclimation. Therefore, data were analyzed for the following time epochs: Light 1 (L1) = 6–10 min, Dark 1 (D1) = 11–15 min, Light 2 (L2) = 16–20 min, and Dark 2 (D2) = 21–35 min. Mixed effects regression models, including zebrafish-specific random effects, were used to assess differences between groups. DMSO groups from each spawn date (≥3 independent spawns) and plate were combined in the analysis. Separate models were fit for each congener. New variables were defined to capture differences in the AUC between Light 1 and Dark 1, Dark 1 and Light 2, and Light 2 and Dark 2, to measure change in distance traveled when transitioning from light to dark or dark to light. Contrasts were specified to compare these transitions and the AUC during each of the lighting conditions between the exposed groups and the DMSO control group. Exploratory analysis indicated that a natural logarithmic transformation was needed for the AUC to stabilize the variance and meet the underlying assumptions of the mixed effects models. Due to some zeroes in the AUC, all values were shifted by 0.1 prior to taking the natural logarithm. Concentration (0.1, 0.3, 1, 3, 10 µM and DMSO), day (3, 4, 5 dpf), and the transition variables were all of interest in the models, including their interactions. Akaike information criterion was used for model selection and Wald tests for comparing groups. All analyses were conducted using SAS version 9.4.

### Extraction of PCBs from zebrafish larvae

PCB body burden was assessed at 5 dpf by pressurized liquid extraction Dionex ASE200 system (Thermo Scientific, Sunnyvale, CA, United States) (Wu et al., 2015). Briefly, zebrafish larvae samples (*n* = 36–51 per sample) were mixed with diatomaceous earth (2 g) and placed in an extraction cell (33 ml) containing Florisil (60–100 mesh, 12 g, Fisher Scientific, Fair Lawn, NJ, United States). PCB 117 (100 ng) was added to each sample as surrogate recovery standard, and the cells were extracted with hexane-acetone (1:1, v/v) at 100°C and 1,500 psi (10 MPa) with preheat equilibration for 6 min, 35% of cell flush volume and 1 static cycle of 5 min. Method blanks containing only Florisil and diatomaceous earth and sample blanks with naïve (unexposed) and/or DMSO-exposed zebrafish larvae were extracted in parallel with each sample set. The extracts were concentrated to approximately 0.75 ml using a Turbo Vap II (Biotage, Charlotte, NC, United States) and transferred to glass tubes. Samples were further cleaned up, as described previously, to remove sulfur and other impurities (Kania-Korwel et al., 2007). All samples were spiked with PCB 204 (100 ng) as internal standard (volume corrector) prior to gas chromatographic analysis.

### Gas chromatography analysis

Analysis of PCBs was performed in the splitless mode on an Agilent 7890 gas chromatograph equipped with a ^63^Ni micro electron capture detector (µECD) and a SPB-1 capillary column (length, 60 m; inner diameter, 250 μm; and 0.25 µm film thickness; Supelco, St. Louis, MO, United States) (Wu et al., 2013). The following temperature program was used based on a published method (Kania-Korwel et al., 2011): hold at 80°C for 1 min, 20°C/min to 215°C, then 0.1°C/min to 219.5°C, and 20°C/min to 280°C, then hold at 280°C for 3 min. A constant helium flow rate of 1 ml/min was used for all analyses. The injector and detector temperatures were 250°C and 300°C, respectively. The ^63^Ni-μECD used for the PCB analysis was linear up to concentrations of 1,000 ng/ml for all analytes investigated (*R* ^2^ > 0.999). The recovery of PCB 117 was 83 ± 7% (range: 71–97%). A detailed summary of the limits of detection, limits of quantification, and background levels of the analyzed PCBs is presented in Supplementary Table S1.

### Enantioselective gas chromatographic analysis of PCB 84 and PCB 95

The enantioselective analysis of PCB 84 was performed on a 6890 Agilent gas chromatograph equipped with a ^63^Ni-µECD detector and CP-Chiralsil-Dex CB capillary column with cyclodextrin directly bonded to dimethylpolysiloxane (25 m length, 0.25 mm inner diameter, 0.25 µm film thickness; Varian, Palo Alto, CA, United States). The temperature program was as follows (Kania-Korwel et al., 2006): starting temperature 50°C, hold for 1 min, 10°C/min to 140°C for 100 min, then 10°C/min to 200°C for 5 min. The enantioselective analysis of PCB 95 was performed on a 7890 Agilent gas chromatograph equipped with a ^63^Ni-µECD detector and Chiraldex B-DM (BDM) capillary column (length, 30 m × inner diameter, 250 μm × 0.12 µm film thickness, Supelco; St. Louis, MO, United States). The following temperature program was used (Uwimana et al., 2016): starting temperature 50°C for 1 min, 10°C/min to 160°C for 90 min, then 10°C/min to 200°C for 10 min. For both instruments, the injector was operated in the splitless mode at 250°C, and the detector temperature was 250°C. A constant helium flow rate of 3 ml/min was used for all enantioselective analyses. Enantiomer fractions (EF) were calculated with the drop valley method (Asher et al., 2009; Uwimana et al., 2016) as EF = Area E_1_/(Area E_1_ + Area E_2_), were Area E_1_ and Area E_2_ denote the peak area of the first and second eluting atropisomer, respectively. The resolution of two atropisomers of PCB 84 and PCB 95 were 0.79 and 1.2, respectively. The EF values were 0.5 for the racemic PCB 84 and PCB 95 standards.

### Neurotoxicity ranking of congeners

The neurotoxic potential of the congeners tested in this study were ranked based on the three endpoints measured in this study: RYR binding, behavior, and body burden. Congeners were ranked relative to each other for each individual endpoint from 1 (highest) to 6 (lowest), and then the three scores for each congener averaged to obtain an overall relative ranking for each congener. Ranking for RYR activation was based on EC_2X_ values and their relative comparisons. Behavior ranking represents the total change from control for each congener across all epochs (L1, D1, L2, and D2) and all days (3, 4, and 5 dpf) tested. AUC values were normalized to the appropriate DMSO control (AUC value/DMSO). Each value was then subtracted from 1 to determine the deviation from the control values [1 − (AUC value/DMSO)]. Absolute values were combined for all concentrations of each congener across all time points (epochs and testing days). PCB body burden was ranked as the percentage uptake using the following calculation: (uptake/total PCB) × 100, where, the uptake values were the measured PCB levels in the samples, and the total PCB values were calculated using the exposure concentration (0.1, 1, or 10 μM) and volume (200 μl). The resulting values were averaged across the three assayed concentrations and rounded to the closest integer to obtain the final ranking order.

## Results

### PCB activation of zebrafish RYR

**FIGURE 1:**
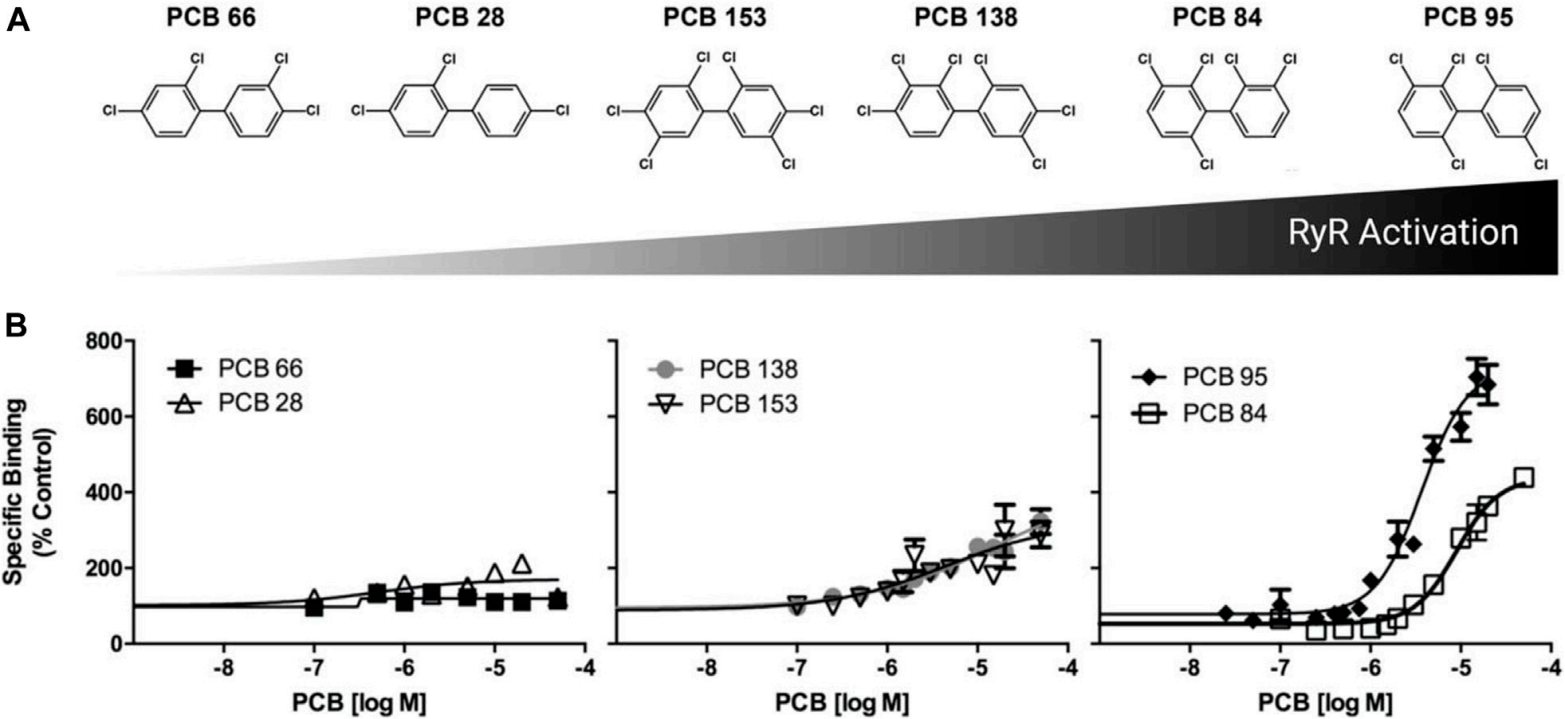
PCB induced [3H]-labeled ryanodine activation and binding in adult zebrafish skeletal muscle. **(A)** Depiction of congener structure and relative RYR activation. **(B)** [^3^H]-ryanodine (Ry) binding was measured in the presence of specific PCB congeners at 0.1–50 µM. Specific binding shown as a percentage of the DMSO control. Data presented as mean ± SEM from six adult (∼1 year old) zebrafish.

Adult zebrafish tail muscle homogenates were used to test RYR binding of PCBs 28, 66, 84, 95, 138, and 153 (Figure 1A). [^3^H]Ry binds with high affinity to the open RYR channel, where increased binding signifies increased channel activity in the presence of PCBs. PCB 28 and 66 displayed limited RYR activity, where even concentrations above 10 µM failed to cause a 2-fold (EC_2X_) increase in channel activity. The environmentally prevalent PCB 138 and 153 presented moderate RYR activity (Figure 1 and Table 1). Interestingly, PCB 84 exhibited a biphasic effect (Supplementary Figure S13) in which concentrations below 3 µM caused a significant reduction in RYR activity. Conversely, concentrations above 3 µM caused modest activation with a maximal effect of 400% compared to the DMSO control. Similar to that seen in mammalian species, PCB 95 was the most efficacious at sensitizing RYR, causing an approximate 800% increase in [^3^H]Ry-binding, a measure of channel activation. Relative RYR activity is summarized in Figure 1B. As predicted, PCBs with less than two chlorines in the *ortho*-position displayed weak or negligible RYR activity. In contrast, congeners with *meta* substitutions adjacent to *ortho*-chlorines (PCB 84, 95, and 138) displayed higher activity at the channel. PCB 95 and 84, which lack *para* substitutions, were the most potent RYR sensitizers.

**TABLE 1:** PCB-induced activation of the RYR paralogs found in zebrafish skeletal muscle.

Congener	Substitution pattern	Max. response SEM (%)	EC_50_	EC_50_ 95% CI	EC_2X_	EC_2X_ 95% CI
PCB 28	2,4,4′	170.60 ± 13.28	0.67	0.08–5.0	-	-
PCB 66	2,3′,4,4′	-	-	—	-	-
PCB 84	2,2′,3,3′,6	437.50 ± 35.72	8.97	6.65–12.08	6.97	5.86–8.24
PCB 95	2,2′,3,5′,6	734.70 ± 49.87	3.79	2.79–5.14	1.5	1.09–1.95
PCB 138	2,2,3,4,4′,5′	388.20 ± 74.93	9.6	2.16–42.97	4.18	3.16–4.64
PCB 153	2,2′,4,4′,5,5′	320.40 ± 92.18	4.34	0.3–58.72	3.88	2.06–8.37

Maximum Response observed as compared to the DMSO control; relative EC50, concentration that caused half-maximum response for a given congener; EC2X, concentration which caused a two-fold induction in ligand binding as compared to the DMSO control. CI, Confidence interval. The hyphen symbol (-) indicates that a value could not be determined because of negligible RYR activity. Hyphen indicates parameters that were not available due to a lack of or low activity of a given congener.

### Viability and teratogenic effects

**FIGURE 2:**
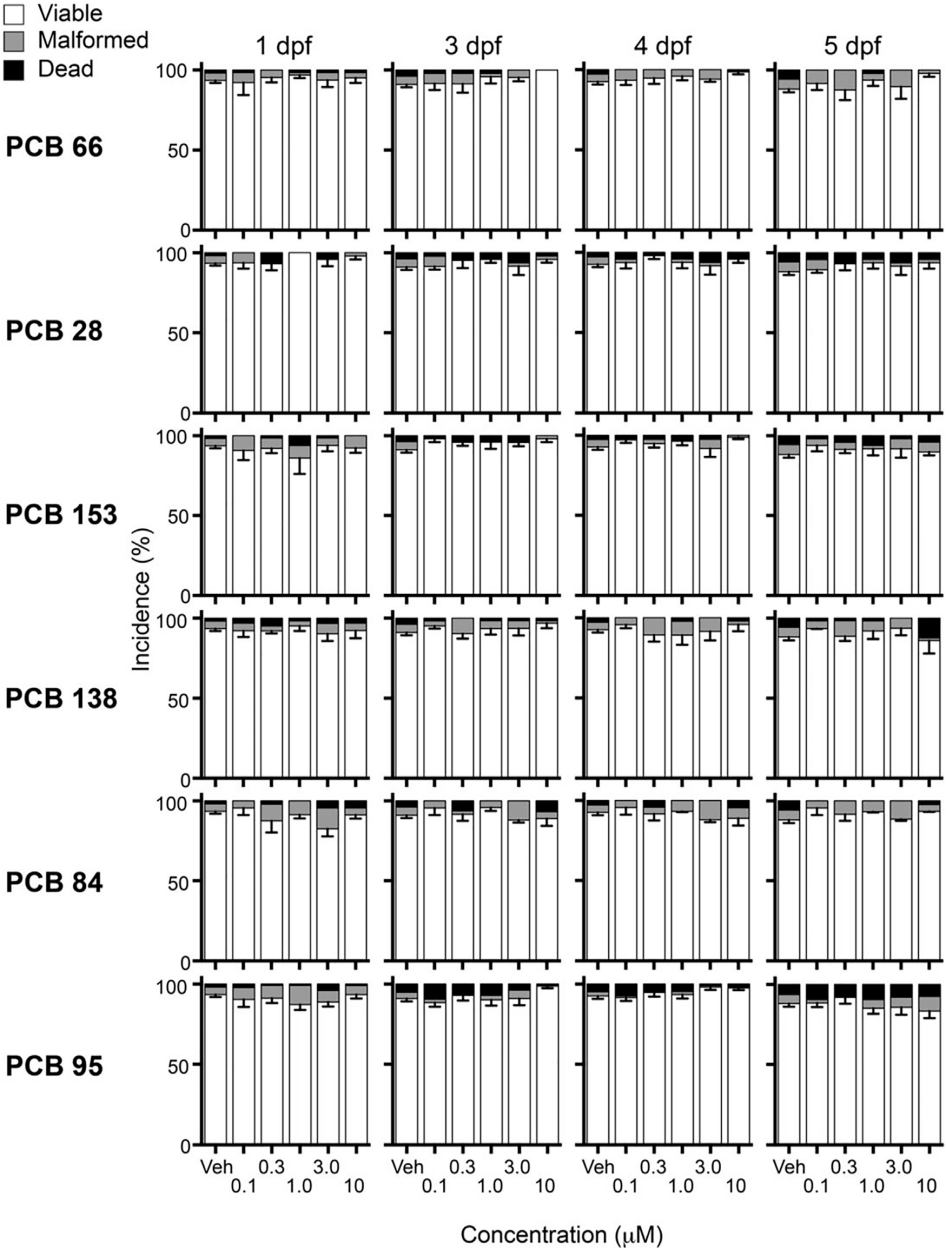
PCB exposures did not significantly decrease viability (congeners listed in order of increasing RYR binding affinity). Larvae were observed for overt malformations or mortalities at 1, 3, 4, and 5 days post-fertilization (dpf). Data are presented as mean percentage (malformed and dead) or mean percentage (viable) ± SEM from ≥3 separate spawns. The total larvae per exposure (Vehicle (Veh), 0.1, 0.3, 1.0, 3.0, and 10 µM) was the following: PCB 66: 32, 60, 61, 63, 60, and 60; PCB 28: 23, 45, 44, 48, 48, and 47; PCB 153: 28, 58, 58, 58, 60, and 60; PCB 138: 28, 59, 61, 63, 59, and 59; PCB 84: 32, 56, 56, 57, 52, and 57; PCB 95: 61, 85, 115, 111, 113, and 119.

Zebrafish were statically exposed to PCBs starting at 6 hpf through 5 dpf and observed daily. Each of the six PCB congeners was tested at the same five concentrations ranging from 0.1 to 10 µM. Within this concentration range, there were no significant changes in zebrafish viability or teratogenic effects (yolk sac or pericardial edema, malformation of caudal fin, pectoral fins, eyes, craniofacial structures, axis, or notochord) of PCB-exposed larvae when compared to the corresponding vehicle control at 1, 3, 4, and 5 dpf (Figure 2). However, exposure to 30 µM PCB 95 resulted in nearly 100% loss of viability by 3 dpf (data not shown). The other PCB congeners were not tested at 30 µM due to solubility constraints.

### Larval behavior—photomotor assay

**FIGURE 3:**
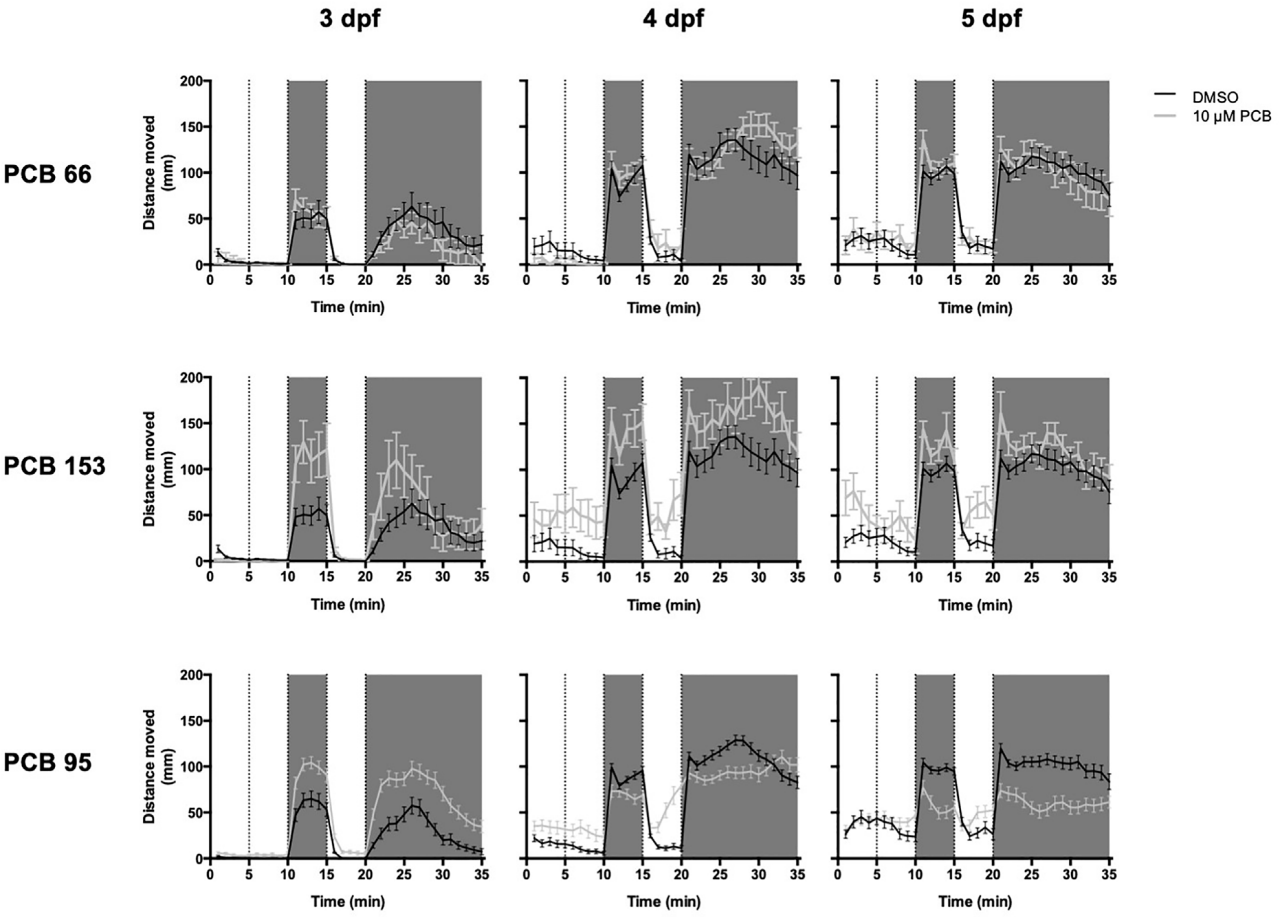
PCB exposure (10 µM) significantly alters larval photomotor behavior at 3 and 5 days post-fertilization (dpf) compared to the vehicle (DMSO) control in a RYR-dependent pattern. Examples of photomotor behavior at 3, 4, and 5 dpf from embryos exposed to only vehicle (DMSO, black lines) or 10 µM (grey line) of PCB 66 (low RYR-activity), PCB 153 (mid-level RYR-activity) and PCB 95 (high RYR-activity). The 35-min behavioral testing period was divided into five epochs: 0–5 min = acclimation period, 5–10 min = Light 1, 11–15 = Dark 1, 16–20 min = Light 2 and 21–35 min = Dark 2. Shaded areas correspond to dark periods. Data are presented as mean ± SEM (*n* = 74, 74, and 55 for PCB 95-exposed embryos and *n* = 75, 75, and 60 for vehicle control embryos on 3, 4, and 5 dpf, respectively).

Vehicle [0.2% (v/v) DMSO] control larvae exhibited increased locomotion in the dark epochs (D1 and D2) and reduced locomotion in the light epochs (L1 and L2), consistent with normal larval photomotor behavior (Basnet et al., 2019). An overall developmental effect was observed in which locomotion in both light and dark epochs increased at 4 dpf compared to 3 dpf and increased at 5 dpf compared to either 4 dpf or 3 dpf (3 dpf < 4 dpf < 5 dpf) (Table 2). To quantify this behavior further, we analyzed light epoch transitions, which are defined as the difference in distance moved from L1-D1, D1-L2, and L2-D2. As expected, in vehicle control larvae there was a significant increase in the distance moved with the L1-D1 (*p* < 0.001) and L2-D2 (*p* < 0.001) transitions and a significant decrease in distance moved with the D1-L2 transition (*p* < 0.001). Furthermore, the transitions between light epochs (L1-D1, D1-L2 and L2-D2) decreased with increased larval age (3 dpf > 4 dpf > 5 dpf) (Figure 3).

**TABLE 2:** Behavioral results at 3, 4, and 5 dpf for PCB-exposed and vehicle control larvae.

Con.	Conc. (μM)	3 dpf				4 dpf				5 dpf			
		L1	D1	L2	D2	L1	D1	L2	D2	L1	D1	L2	D2
28	VEH	−1.4 (0.1)	4.3 (0.2)	0.05 (0.2)	4.0 (0.2)	0.4 (0.3)	5.3 (0.2)	2.7 (0.2)	6.5 (0.1)	2.7 (0.2)	5.8 (0.03)	3.3 (0.1)	6.7 (0.1)
	0.1	−1.3 (0.2)	4.2 (0.3)	0.2 (0.2)	4.1 (0.2)	−0.03 (0.3)	5.9 (0.1)	2.6 (0.2)	6.7 (0.1)	2.7 (0.3)	5.8 (0.04)	3.1 (0.2)	6.7 (0.1)
	0.3	−1.2 (0.2)	4.3 (0.2)	0.02 (0.2)	3.8 (0.3)	−0.4 (0.3)	5.9 (0.05)	2.8 (0.2)	6.6 (0.1)	2.6 (0.3)	5.8 (0.03)	3.2 (0.2)	6.7 (0.1)
	1	−1.3 (0.2)	4.6 (0.2)	0.1 (0.2)	3.6 (0.3)	−0.04 (0.3)	5.9 (0.05)	2.4 (0.2)	6.7 (0.1)	2.7 (0.3)	5.9 (0.05)	3.3 (0.1)	6.8 (0.1)
	3	−1.1 (0.2)	4.4 (0.2)	0.2 (0.2)	3.6 (0.3)	−0.1 (0.3)	5.9 (0.04)	2.5 (0.3)	6.7 (0.1)	2.8 (0.3)	5.8 (0.04)	3.3 (0.2)	6.7 (0.1)
	10	−1.5 (0.2)	3.9 (0.2)	0.3 (0.2)	2.9 (0.3)	−0.05 (0.3)	5.9 (0.1)	1.9 (0.2)	6.3 (0.2)	2.5 (0.3)	6.0 (0.05)	2.5 (0.2)	6.5 (0.1)
66	VEH	−0.6 (0.2)	3.6 (0.2)	0.4 (0.2)	3.6 (0.3)	0.02 (0.3)	5.8 (0.04)	2.6 (0.2)	6.7 (0.1)	2.2 (0.3)	5.9 (0.04)	3.1 (0.2)	6.9 (0.1)
	0.1^a^	−1.2 (0.2)	3.3 (0.3)	0.2 (0.2)	3.2 (0.3)	−0.7 (0.3)	5.8 (0.1)	2.6 (0.2)	6.8 (0.1)	2.1 (0.4)	5.8 (0.05)	3.0 (0.3)	6.8 (0.1)
	0.3	−1.3 (0.2)	3.8 (0.3)	0.5 (0.2)	4.0 (0.3)	−0.4 (0.3)	5.8 (0.05)	2.6 (0.2)	6.9 (0.1)	3.0 (0.3)	5.8 (0.04)	3.4 (0.2)	6.9 (0.1)
	1^a^	−1.1 (0.2)	3.4 (0.3)	0.03 (0.2)	3.2 (0.4)	−1.2 (0.2)	5.8 (0.1)	2.4 (0.2)	6.8 (0.1)	1.7 (0.4)	5.8 (0.05)	3.3 (0.2)	6.8 (0.1)
	3	−1.1 (0.2)	3.6 (0.3)	0.4 (0.2)	3.7 (0.4)	−0.3 (0.3)	5.8 (0.1)	2.5 (0.2)	6.8 (0.1)	2.0 (0.3)	5.8 (0.04)	3.1 (0.2)	6.8 (0.1)
	10	−1.1 (0.2)	3.7 (0.3)	0.3 (0.2)	3.6 (0.4)	−0.6 (0.3)	5.8 (0.1)	3.0 (0.2)	6.8 (0.1)	2.2 (0.4)	5.7 (0.1)	3.4 (0.2)	6.5 (0.1)
84	VEH	−1.2 (0.2)	3.7 (0.2)	0.4 (0.1)	3.7 (0.3)	0.1 (0.3)	5.9 (0.04)	2.9 (0.2)	6.8 (0.8)	2.0 (0.3)	5.9 (0.04)	3.3 (0.1)	6.9 (0.1)
	0.1	−1.2 (0.2)	3.3 (0.3)	0.2 (0.2)	2.7 (0.4)	−0.5 (0.3)	5.9 (0.05)	2.2 (0.3)	6.7 (0.1)	1.6 (0.4)	5.8 (0.1)	3.0 (0.2)	6.8 (0.1)
	0.3	−1.1 (0.2)	3.9 (0.3)	0.3 (0.2)	3.7 (0.3)	−0.8 (0.3)	5.8 (0.04)	2.9 (0.2)	6.7 (0.1)	2.7 (0.4)	5.9 (0.04)	3.4 (0.2)	6.9 (0.1)
	1	−0.7 (0.2)	3.8 (0.3)	0.3 (0.2)	3.5 (0.3)	−0.9 (0.3)	5.8 (0.1)	2.6 (0.2)	6.8 (0.1)	2.1 (0.3)	5.8 (0.04)	3.2 (0.2)	6.8 (0.1)
	3	−0.8 (0.2)	4.0 (0.3)	0.4 (0.2)	4.0 (0.3)	−0.1 (0.3)	5.8 (0.04)	3.6 (0.2)	6.8 (0.1)	3.8 (0.3)	5.6 (0.1)	3.4 (0.2)	6.5 (0.1)
	10	−0.4 (0.3)	4.7 (0.2)	1.3 (0.2)	5.0 (0.2)	0.7 (0.4)	5.6 (0.1)	3.3 (0.3)	6.7 (0.1)	2.7 (0.4)	5.4 (0.1)	3.6 (0.3)	6.3 (0.1)
95	VEH	−0.9 (0.1)	4.4 (0.2)	0.7 (0.1)	4.7 (0.2)	0.3 (0.2)	5.4 (0.2)	3.0 (0.1)	7.2 (0.05)	2.5 (0.2)	6.0 (0.03)	3.6 (0.1)	7.2 (0.04)
	0.1	−0.5 (0.3)	4.3 (0.3)	0.6 (0.2)	4.6 (0.4)	−0.4 (0.4)	5.6 (0.2)	3.1 (0.3)	7.0 (0.2)	3.0 (0.4)	5.8 (0.05)	3.8 (0.2)	7.1 (0.1)
	0.3	−0.6 (0.2)	4.6 (0.2)	0.6 (0.2)	4.7 (0.2)	0.4 (0.3)	5.0 (0.2)	3.1 (0.2)	7.2 (0.1)	2.7 (0.3)	5.9 (0.05)	3.8 (0.2)	7.2 (0.05)
	1	−0.8 (0.2)	4.4 (0.2)	0.8 (0.2)	4.4 (0.2)	−0.004 (0.3)	5.0 (0.3)	3.3 (0.2)	7.3 (0.05)	2.5 (0.3)	5.9 (0.04)	3.8 (0.2)	7.3 (0.05)
	3	−0.7 (0.2)	4.8 (0.2)	0.9 (0.2)	5.1 (0.2)	0.009 (0.3)	5.2 (0.2)	3.0 (0.2)	7.3 (0.05)	3.7 (0.3)	5.8 (0.04)	4.2 (0.2)	7.2 (0.05)
	10	0.6 (0.3)	5.7 (0.1)	2.7 (0.2)	6.5 (0.1)	2.0 (0.3)	4.7 (0.3)	4.2 (0.2)	7.1 (0.1)	4.2 (0.3)	4.9 (0.2)	4.3 (0.2)	6.2 (0.2)
138	VEH	−0.9 (0.1)	3.7 (0.2)	0.08 (0.1)	3.6 (0.2)	0.05 (0.2)	5.8 (0.1)	2.6 (0.1)	6.7 (0.1)	2.3 (0.2)	5.9 (0.03)	3.0 (0.1)	6.7 (0.1)
	0.1	−0.9 (0.2)	3.8 (0.3)	0.02 (0.2)	3.5 (0.3)	−0.6 (0.3)	5.6 (0.1)	2.3 (0.2)	6.4 (0.1)	2.5 (0.3)	5.6 (0.1)	3.1 (0.2)	6.5 (0.1)
	0.3	−1.4 (0.1)	4.0 (0.3)	−0.1 (0.2)	3.7 (0.3)	−0.6 (0.3)	5.9 (0.04)	2.8 (0.2)	6.7 (0.1)	2.1 (0.3)	5.9 (0.05)	3.1 (0.2)	6.8 (0.1)
	1	−0.9 (0.2)	4.2 (0.2)	0.2 (0.2)	3.9 (0.3)	−0.6 (0.3)	5.8 (0.05)	2.8 (0.2)	6.7 (0.1)	2.7 (0.4)	5.8 (0.05)	3.1 (0.2)	6.7 (0.1)
	3	−0.7 (0.2)	4.3 (0.2)	0.2 (0.2)	4.3 (0.2)	0.02 (0.3)	5.8 (0.05)	2.7 (0.2)	6.6 (0.1)	2.9 (0.3)	5.7 (0.1)	3.7 (0.2)	6.7 (0.1)
	10	−0.5 (0.2)	4.9 (0.2)	0.6 (0.2)	4.6 (0.2)	0.9 (0.3)	6.0 (0.05)	3.0 (0.2)	6.7 (0.1)	2.3 (0.3)	5.4 (0.1)	3.4 (0.2)	6.0 (0.1)
153	VEH	−0.7 (0.2)	4.3 (0.3)	0.3 (0.2)	4.4 (0.3)	0.1 (0.3)	5.9 (0.04)	2.4 (0.2)	6.8 (0.1)	2.5 (0.3)	5.9 (0.04)	3.0 (0.2)	6.8 (0.1)
	0.1	−1.4 (0.2)	4.4 (0.3)	0.1 (0.3)	4.5 (0.3)	0.8 (0.5)	5.9 (0.05	2.8 (0.2)	6.9 (0.1)	2.7 (0.4)	5.8 (0.05)	3.3 (0.3)	6.8 (0.1)
	0.3	−1.1 (0.2)	4.5 (0.3)	0.3 (0.2)	4.1 (0.3)	1.0 (0.5)	5.9 (0.05)	2.6 (0.3)	6.8 (0.1)	1.9 (0.4)	5.8 (0.04)	3.1 (0.3)	6.9 (0.1)
	1	−1.2 (0.2)	5.0 (0.2)	0.6 (0.2)	4.8 (0.3)	0.5 (0.4)	5.9 (0.05)	3.1 (0.3)	6.9 (0.1)	2.8 (0.4)	5.9 (0.05)	3.5 (0.2)	6.9 (0.1)
	3	−1.2 (0.2)	4.7 (0.2)	0.4 (0.3)	4.4 (0.3)	0.1 (0.4)	5.9 (0.05)	2.8 (0.2)	6.8 (0.1)	2.7 (0.4)	5.7 (0.1)	3.1 (0.2)	6.8 (0.1)
	10	−1.1 (0.2)	5.4 (0.3)	1.4 (0.2)	5.5 (0.3)	0.8 (0.5)	6.1 (0.1)	3.7 (0.3)	7.0 (0.1)	3.4 (0.3)	6.1 (0.1)	4.1 (0.3)	6.8 (0.1)

Data are presented as the mean of the natural log of the area under the curve (AUC) with the standard error in parentheses from each period for each concentration and vehicle (VEH) controls used for comparison. Color-coding of the cells is used to signify increases (orange) or decreases (blue) in distance traveled relative to VEH. The significant differences were determined using a mixed-effects model that considers the spread and distribution of the data and are outlined in the *Materials and Methods* section. Larvae were scored for viability following behavioral testing and non-viable individuals were removed from the data set prior to analysis. Values shown in bold type are significantly different from corresponding vehicle control, using the mixed effects regression model as described in the text.

a The best model for congener 66 did not suggest that differences in how behavior at various concentrations compared to vehicle controls varied by larval age. However, on average, across the ages, distance traveled was lower, on average, during L1 in the 0.1 and 1 concentrations compared to vehicle controls. Light 1 (L1) = 5 min, Dark 1 (D1) = 5 min, Light 2 (L2) = 5 min and Dark 2 (D2) = 15 min.

Exposure to PCB 66 had the least impact on behavior in the photomotor assay. Exposure to 0.11 µM (*p* = 0.03) or 1 µM (*p* = 0.001) PCB 66 significantly decreased distance traveled in L1 relative to age-matched vehicle control larvae, on average, across larval ages (*p* < 0.03; Figure 3 and Table 2), and the difference did no significantly vary across age. The other four PCB congeners had a greater impact on photomotor behavior with significant effects observed at multiple concentrations with respect to both distance moved and transitions between light epochs. The results for distance traveled are summarized in Table 2 and the transition findings are summarized in Supplementary Figures S14–S19.

At 3 dpf, larvae exposed to 3 and 10 µM PCB 28 traveled significantly less distance in D2 than vehicle control larvae (*p* = 0.049 and *p* < 0.001, respectively). At 4 dpf, all PCB 28-exposed larvae, except for those exposed to 10 μM, translocated significantly more than the vehicle controls in D1 (0.1 µM: *p* = 0.02; 0.3 µM: *p* = 0.005; 1 µM: *p* = 0.03; 3 µM: *p* = 0.03), with those exposed to 0.3 µM traveling less in L1 than vehicle controls (*p* = 0.01). The L1-D1 transition also significantly increased relative to vehicle controls [0.1 µM moving 0.9 mm more (*p* = 0.009), 0.3 µM moving 1.3 mm more (*p* < 0.001), 1 µM moving 0.87 mm more (*p* = 0.009), 3 µM moving 0.9 (*p* = 0.009) and 10 µM moving 0.8 more mm (*p* = 0.02)] (Supplementary Figure S15). At 5 dpf, larvae exposed to 10 µM moved less than vehicle controls in L2 (*p* = 0.048).

Similarly, at 3 dpf, larvae exposed to 1 or 10 µM PCB 153 moved more in D1, D2, and L2 than vehicle controls (*p* < 0.03); the L1-D1 transition was larger in the larvae exposed to 1 µM or 10 µM PCB 153 than vehicle controls (*p* < 0.025). However, at 4 dpf, larvae exposed to 0.3 µM PCB 53 moved more than vehicle controls in L1 (*p* = 0.02), attenuating the L1-D1 transition (*p* = 0.046), while larvae exposed to 10 µM PCB 153 moved more than vehicle controls in L2 (*p* < 0.01). At 5 dpf, larvae exposed to 10 µM PCB 153 moved more in L1 than vehicle controls (*p* = 0.02), which resulted in larvae in an attenuated L1-D1 transition compared to controls (*p* = 0.06, Supplementary Figure S16); this group also exhibited more movement in L2 than controls (*p* = 0.002).

Increased distanced moved in L1 and L2 compared to controls was observed in 3 dpf larvae exposed to PCB 84 at 10 µM (*p* < 0.02), while 3 dpf larvae exposed to 3 µM PCB 84 moved more in L2 (*p* = 0.04) than controls. PCB 138 and PCB 84-exposed larvae also exhibited significantly increased distance moved in D1 and D2 at 3 dpf. Specifically, for PCB 138, the 1 µM (*p* = 0.03), 3 µM (*p* = 0.002) and 10 µM (*p* < 0.001) exposure groups moved more than the vehicle controls in D1 while the 3 µM (*p* < 0.001) and 10 µM (*p* < 0.001) exposure groups moved more than vehicle controls in D2. For PCB 84, the 10 µM exposure group moved more (*p* < 0.001) in D1 and D2 than the age-matched controls. However, there were also differing effects of these two congeners on photomotor behavior. For PCB 84, the 0.1 µM exposure group moved less in D1, L2 and D2 at 3 dpf (D1: *p* = 0.01; L2: *p* = 0.003; D2: *p* < 0.001). PCB 84 exposed larvae also had altered movement at 4 dpf, with the 0.3 and 1 µM exposure groups exhibiting significantly less movement in L1 (*p* = 0.004 and *p* = 0.002, respectively). At 5 dpf, the 0.3, 3, and 10 µM exposure groups moved more in L1 (*p* = 0.047, *p* < 0.001 and *p* = 0.02, respectively). In contrast, 5 dpf larvae exposed to PCB 138 at 10 µM moved less in D2, than the vehicle control fish (*p* = 0.004).

The transition results followed a pattern similar that observed for distance moved amongst larvae exposed to PCB 138 or 84. For PCB 138, at 4 dpf, the L1-D1 transition was increased in the 0.3 µM group (0.821 more; *p* = 0.01) and the 1 µM group (0.68 more; *p* = 0.04) and decreased in the 10 µM group though not quite statistically significant (0.6 less; *p* = 0.06) compared to the control group (Supplementary Figure S17). At 5 dpf, the 3 µM group moved less during the L1-D1 transition compared to controls (0.7 less; *p* = 0.03). For PCB 84 at 4 dpf, the 0.3 and 1 µM groups moved more in the L1-D1 transition (*p* = 0.007 and *p* = 0.005, respectively), while the 10 µM group moved less during the transition (*p* = 0.04) (Supplementary Figure S18). At 5 dpf, the PCB 84 3 and 10 µM groups increased less than the DMSO group in the L1-D1 transition (*p* < 0.001 and *p* = 0.004, respectively).

Larvae exposed to 10 µM PCB 95 moved significantly more in D1 and D2 at 3 dpf (*p* < 0.001 for both) and significantly less at 4 dpf (in D1; *p* = 0.004) and 5 dpf (in both D1 and D2; *p* < 0.001) than vehicle controls (Figure 3). During light conditions, larvae exposed to 10 µM PCB 95 moved significantly more in L1 and L2 at 3 dpf (*p* < 0.001) and 4 dpf (*p* < 0.001) and more in L1 at 5 pdf (*p* < 0.001) than age-matched controls. Larvae exposed to 3 µM moved more in L2 and D2 at 3 dpf (*p* < 0.02) and more in L1 at 5 dpf (*p* < 0.001) than controls. As shown in Supplementary Figure S19, the 3 µM PCB 95 exposure group moved significantly less than DMSO in the L1-D1 transition at 5 dpf (*p* < 0.001), while in the 10 µM PCB 95 exposure group, the L1-D1 transition was decreased on 4 and 5 dpf (*p* < 0.001). In contrast, the larvae exposed to 0.1 µM PCB 95 moved more during the L1-D1 transition at 4 dpf (*p* = 0.04) than vehicle controls.

### PCB levels in larvae

**FIGURE 4:**
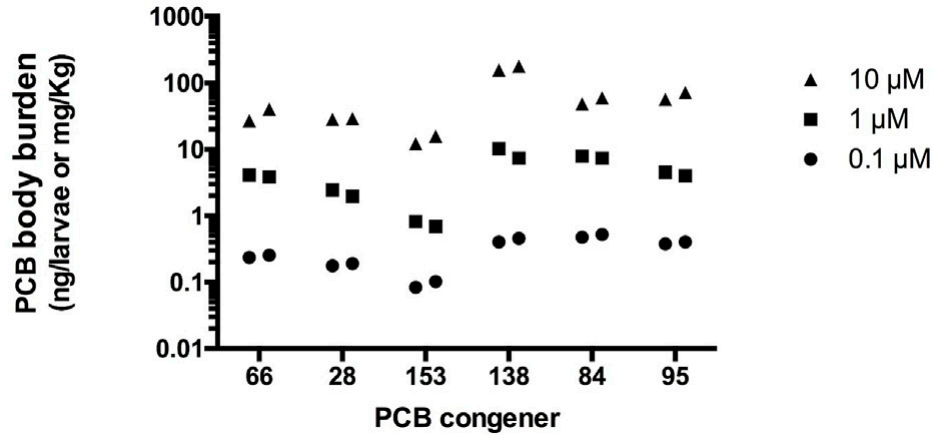
PCB levels in pooled whole fish at 5 dpf exposed to PCB starting at 6 hpf. Each point represents the average body burden in ng per pooled sample of 36–51 larvae collected at 5 dpf. Wet weight per pool is approximately 1 mg, allowing for the extrapolation to mg/kg.

PCB body burden was measured in duplicate for three exposure concentrations (0.1, 1, and 10 µM) of each congener. Exposure began at 6 hpf and tissues were harvested at 5 dpf. For all five PCB congeners, larval body burden was found to increase with increasing PCB concentration (Figure 4). Uptake of PCB 153 was the lowest followed by PCB 28 and PCB 66. PCBs 84 and 95 had comparable body burden, while PCB 138 exposed larvae possessed the highest body burden of the tested PCBs.

Enantioselective GC analysis of PCB 84 and PCB 95 was used to assess possible enantioselective uptake. No significant atropisomeric enrichment of either PCB congener at the concentrations investigated was found, with average EF values of 0.50 ± 0.01 (*n* = 6) and 0.50 ± 0.01 (*n* = 14) for PCB 84 and PCB 95, respectively.

### Congener ranking

**FIGURE 5:**
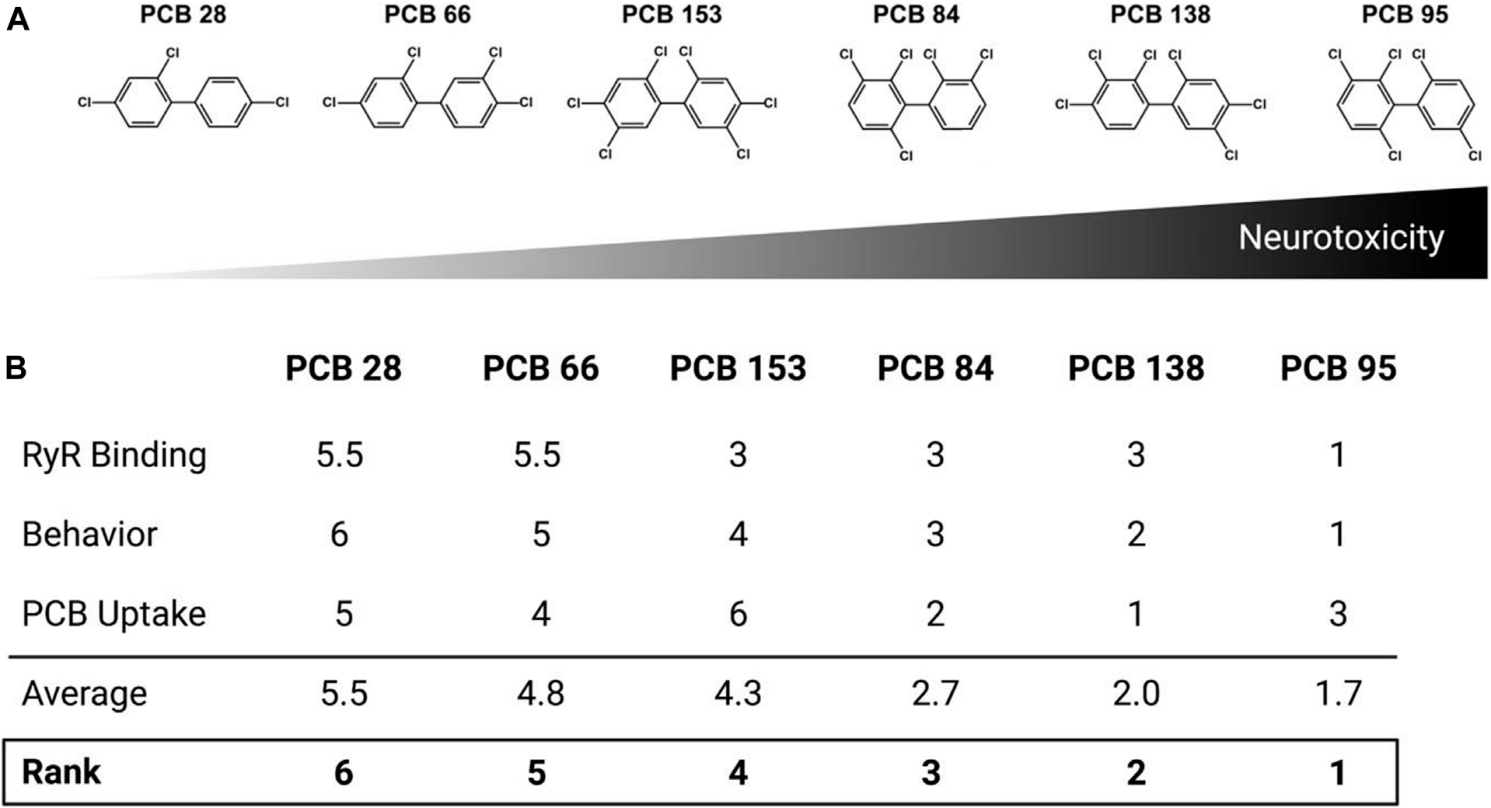
Congener rankings. Rankings were assigned to each congener based on the average ranking from three assays: RYR binding, behavior and body burden. **(A)** Relative congener neurotoxicity in the larval zebrafish with PCB 95 being the most potent. **(B)** Individual scores for each assay, average ranking and overall ranking.

Rankings were assigned to each congener based on the average results of ryanodine binding, photomotor behavior, and PCB body burden. The relative ranking for RYR sensitization (Figure 1) was 66 < 28 < 153 < 138 < 84 < 95. Behavior scores differed noticeably between PCB 95 and the five other congeners: PCB 28 = 10.83 < PCB 66 = 14.39 < PCB 153 = 23.05 < PCB 84 = 28.93 < PCB 138 = 29.32 < PCB 95 = 81.46. To determine the relative ranking for PCB body burden, the % uptake values (measured uptake/theoretical uptake × 100) were averaged across the three concentrations to which zebrafish were exposed: PCB 153 = 1.4% < PCB 28 = 4.5% < PCB 66 = 5.6% < PCB 95 = 7.4% < PCB 84 = 9.2% < PCB 138 = 13.8%. The absolute relative rankings for each endpoint (from 1 to 6) were averaged to determine the relative ranking of the overall congener neurotoxic potency in this zebrafish model: PCB 28 < PCB 66 < PCB 153 < PCB 84 < PCB 138 < PCB 95 (Figure 5). Interestingly, the overall ranking of PCB congeners for potential neurotoxic outcomes follows a similar pattern of their potency in sensitizing the RYR.

## Discussion

PCBs remain an important and persistent pollutant class impacting both wildlife and human health (Beyer and Biziuk 2009; Pessah et al., 2019) and are especially a concern in the context of DNT. Experimental evidence suggests that PCB DNT is largely mediated by NDL PCBs (Klocke and Lein 2020). The most sensitive molecular target of NDL PCBs identified to date is the RYR (Pessah et al., 2010; Pessah et al., 2019).

*In vitro* SAR studies have established the relative potency of NDL PCB congeners for RYR sensitization (Pessah et al., 2006; Fritsch and Pessah 2013; Holland et al., 2017a). While *in vivo* data show that PCBs alter neurodevelopmental endpoints (Kenet et al., 2007; Yang et al., 2009; Yang and Lein 2010; Wayman et al., 2012b) known to be modulated *in vitro* by PCBs via RYR-dependent mechanisms (Howard et al., 2003; Wayman et al., 2012b), there is yet no *in vivo* evidence causally linking PCB sensitization of RYR to PCB DNT. Directly testing RYR involvement in PCB DNT *in vivo* has proven challenging because of technical hurdles, including the lack of pharmacological antagonists or agonists specific for individual RYR isoforms. There are non-specific agonists/antagonists that bind all isoforms (i.e., caffeine), but these often require millimolar concentrations to elicit their effect on RYR and thus often have off-target effects. Genetic knockouts of various RYR isoforms in rodent models are embryonically lethal (Takeshima et al., 1994; Buck et al., 1997; Takeshima et al., 1998) or impact neurobehavioral function (Matsuo et al., 2009). Therefore, in this study we indirectly assessed RYR involvement by determining whether the *in vivo* DNT potency of various NDL PCBs with differential RYR activity parallels their potency in sensitizing the RYR. We tested the specific hypothesis that the SAR for PCB effects on RYR sensitization would predict *in vivo* DNT potency. The findings of our study, while not conclusive proof of RYR-dependent mechanism(s) of PCB DNT, support this hypothesis.

RYR binding studies were first conducted to show whether the PCBs chosen in this study bind to zebrafish RYR and whether their relative sensitization correlates with mammalian *in vitro* findings. Ryanodine preferentially binds to the open configuration of RYR, thus, increased binding of this ligand indicates a greater percentage of channels stabilized in the open configuration. As predicted by SAR studies with mammalian RYR, PCBs with less than two chlorines in the *ortho*-position (PCB 28 and 66) have negligible activity at the RYR. While PCB 66 has a *meta*-chlorine, it is not adjacent to an *ortho*-chlorine, thus confirming previous findings with mammalian RYR that adjacent *ortho*- and *meta*-chlorine substitutions increase RYR potency. The number of *ortho*-chlorines primarily drives RYR sensitization potency. Thus, it is not surprising that there is not a large difference between PCB 138 and 153, which have the same number of chlorines, but the latter has *meta*-chlorines that are not adjacent to *ortho*-chlorines. PCB 84 highlights the importance of *para*-chlorines in determining PCB effects on RYR sensitization. It has the same number of chlorine substitutions as PCB 138 and 153, but PCB 84’s RYR activity is much higher than either PCB 138 or 153 (Table 1), which lack *para* substitutions. Finally, PCB 95 is highly potent in sensitizing zebrafish RYR, which is in agreement with mammalian data (Pessah et al., 2006; Holland et al., 2017a). We used adult zebrafish caudal muscle to conduct the binding studies, as it is the most abundant source of tissue to isolate RYR1 paralogs. In mammalian models, RYR1 and RYR2 dominate RYR expression in the brain and the SAR of PCBs in sensitizing the RYR are similar for these two RYR isoforms (Holland et al., 2017b). These data, which are the first description of the SAR of NDL PCBs towards RYR sensitization in larval zebrafish, are consistent with previously published work in the trout model (Fritsch and Pessah, 2013).

Based on our teratological assessment, the PCB exposures examined in this study were not overtly toxic as evidenced by the lack of any gross morphological abnormalities in exposed zebrafish. Our teratogenic results are consistent with previously published work that reported no malformations or mortality in larval zebrafish younger than 7 dpf statically exposed to PCB 153 at concentrations as high as 10 μM (Aluru et al., 2020). However, our results differ from other studies that showed developmental exposures to PCB 95 caused malformations (Ranasinghe et al., 2020). This discrepancy may reflect the different exposure paradigms employed between studies. In the previous study in which PCB 95 was observed to have teratogenic effects, exposure to PCB 95 commenced shortly after fertilization, whereas our exposures started at 6 hpf, when cell fate has been determined and gastrulation has commenced (Kimmel et al., 1995). Thus, the concentrations and timing of exposure we have chosen in this study are ideal for assessing DNT through the apical endpoint of behavioral assessment.

It has previously been demonstrated in the zebrafish model that RYR activity affects gene expression patterns and consequently cell fates early in development through sonic hedgehog signaling. Dysregulation of RYR-dependent calcium signals disrupted tissue patterning, affecting precursor cells of the nervous system, craniofacial structures, and limbs (Klatt Shaw et al., 2018). While these studies relied on knockout lines, we used pharmacological tools to stabilize the RYR in the open configuration, which has been shown in mammalian systems to perturb calcium signaling (Wayman et al., 2012a). As noted, our exposure concentrations did not affect gross morphology or body patterning but did alter photomotor behavior. The SAR of PCB effects on photomotor behavior closely mimicked the potency ranking of RYR sensitization. Larva exposed to PCBs that more strongly sensitized the RYR exhibited more severe behavioral deficits in the photomotor assay as evidenced by altered swimming behavior in more than one light cycle on more than 1 day of behavioral testing. Interestingly, PCB 28, which was one of the least active congeners in terms of RYR sensitization, caused larva to exhibit hypoactive behavior in the last dark epoch at 3 dpf but hyperactive behavior in the first dark epoch at 4 dpf with minimal behavioral abnormalities observed at 5 dpf. Exposure to congeners with more potent RYR sensitizing activity resulted in hyperactive swimming during dark cycles at 3 dpf that switched to hypoactivity in the dark cycles but hyperactivity in the light cycles with an absence of acclimation to the dark (D2). The results at 5 dpf specifically show decreased AUC during dark epochs (most strongly seen in D2) and increased AUC during light epochs compared to controls. However, at 5 dpf, which epochs were found to be statistically significantly different from controls varied, e.g., for PCB 84, statistically significant differences for AUC were observed in L1, apparent as an increase, while for PCB 95, statistically significant differences were observed in D1 and D2, apparent as a decrease. In summary, for PCBs 84 and 95, the behavioral pattern observed at 4 and 5 dpf manifested as continuous swimming across light transitions, implying loss of sensitivity towards light/dark stimuli. While the relevance of these phenotypes to human DNT has yet to be established, the results underscore the importance of screening behavioral outcomes at multiple developmental stages, especially considering that most zebrafish larval behavioral testing is conducted at 5 dpf (Dach et al., 2019). Whether PCB-induced changes in photomotor behavior reflect PCB effects on RYRs expressed in skeletal muscle, at neuromuscular junctions, peripheral sensory organs involved in light detection, or the central nervous system is unknown. However, if PCBs only affected RYRs in the skeletal muscle, likely one of two behavioral phenotypes would have been observed: 1) consistent hyperactive behavior due to constant activation of the channel or 2) consistent hypoactive behavior due to depletion of calcium stores. However, we did not observe either of those phenotypes in our results.

To confirm that the differential effects between PCB congeners were not simply the result of differential uptake into fish tissues, the body burden of each PCB was determined. The uptake was concentration-dependent for all congeners. Interestingly, the body burdens did not appear to follow patterns of chlorine placement or congener weight. Moreover, the non-racemic PCB 84 and PCB 95 residues suggest that PCBs were not metabolized to an appreciable extent in our model system. In contrast, PCBs can undergo metabolism in mammals, resulting in potentially RYR-active PCB metabolites and, in the case of PCB 84 and PCB 95, a species-dependent enantiomeric enrichment that can affect RYR dependent endpoints *in vitro*. The PCB-induced effects on larval behavior were independent from the body burden. However, as the uptake between congeners differed, we included them in our scoring to account for the overall rank.

In summary, this study demonstrated that the SAR for NDL PCB disruption of photomotor behavior paralleled the SAR for sensitization of the RYR in zebrafish, supporting RYR sensitization as a mechanism contributing to the *in vivo* DNT of NDL PCBs. Evidence supporting a role for RYR-dependent mechanisms of PCB DNT is critical for establishing mechanistic screening tools for DNT and suggests that individuals with heritable mutations in RYR or other molecules involved in Ca^2+^-dependent signaling may be at increased risk for adverse neurodevelopmental outcomes following early life exposures to NDL PCBs.

## Data Availability

The original contributions presented in the study are included in the article/Supplementary Material, further inquiries can be directed to the corresponding author.
