# Supplementary material for: Ryanodine receptor-active non-dioxin-like polychlorinated biphenyls cause neurobehavioral deficits in larval zebrafish
Source: Front Toxicol. 2022 Oct 6;4:947795. doi: 10.3389/ftox.2022.947795 (PMC9582434; doi:10.3389/ftox.2022.947795)
Supplement: Supplementary file 1 [file DataSheet1.pdf]

Supplementary Material

Table of contents

Figure S1 GC-MS chromatogram and mass spectrum of PCB 28 in TIC mode

Figure S2 Gas chromatogram of PCB 28 in SIM mode

Figure S3 Gas chromatogram and mass spectrum of PCB 66 in TIC mode

Figure S4 Gas chromatogram of PCB 66 in SIM mode

Figure S5 Gas chromatogram and mass spectrum of PCB 84 in TIC mode

Figure S6 Gas chromatogram of PCB 84 in SIM mode

Figure S7 Gas chromatogram and mass spectrum of PCB 95 in TIC mode

Figure S8 Gas chromatogram of PCB 95 in SIM mode

Figure S9 Gas chromatogram and mass spectrum of PCB 138 in TIC mode

Figure S10 Gas chromatogram of PCB 138 in SIM mode

Figure S11 Gas chromatogram and mass spectrum of PCB 153 in TIC mode

Figure S12 Gas chromatogram of PCB 153 in SIM mode

Figure S13 RyR activity of PCB 84

Figure S14 PCB 66 behavioral results

Figure S15 PCB 28 behavioral results

Figure S16 PCB 153 behavioral results

Figure S17 PCB 138 behavioral results

Figure S18 PCB 84 behavioral results

Figure S19 PCB 95 behavioral results

Table S1 Limits of detection (*LOD*s), limits of quantification (*LOQ*s), background levels of PCBs in untreated Zebrafish larvae, and recovery rate of method spike and sample spike.

References

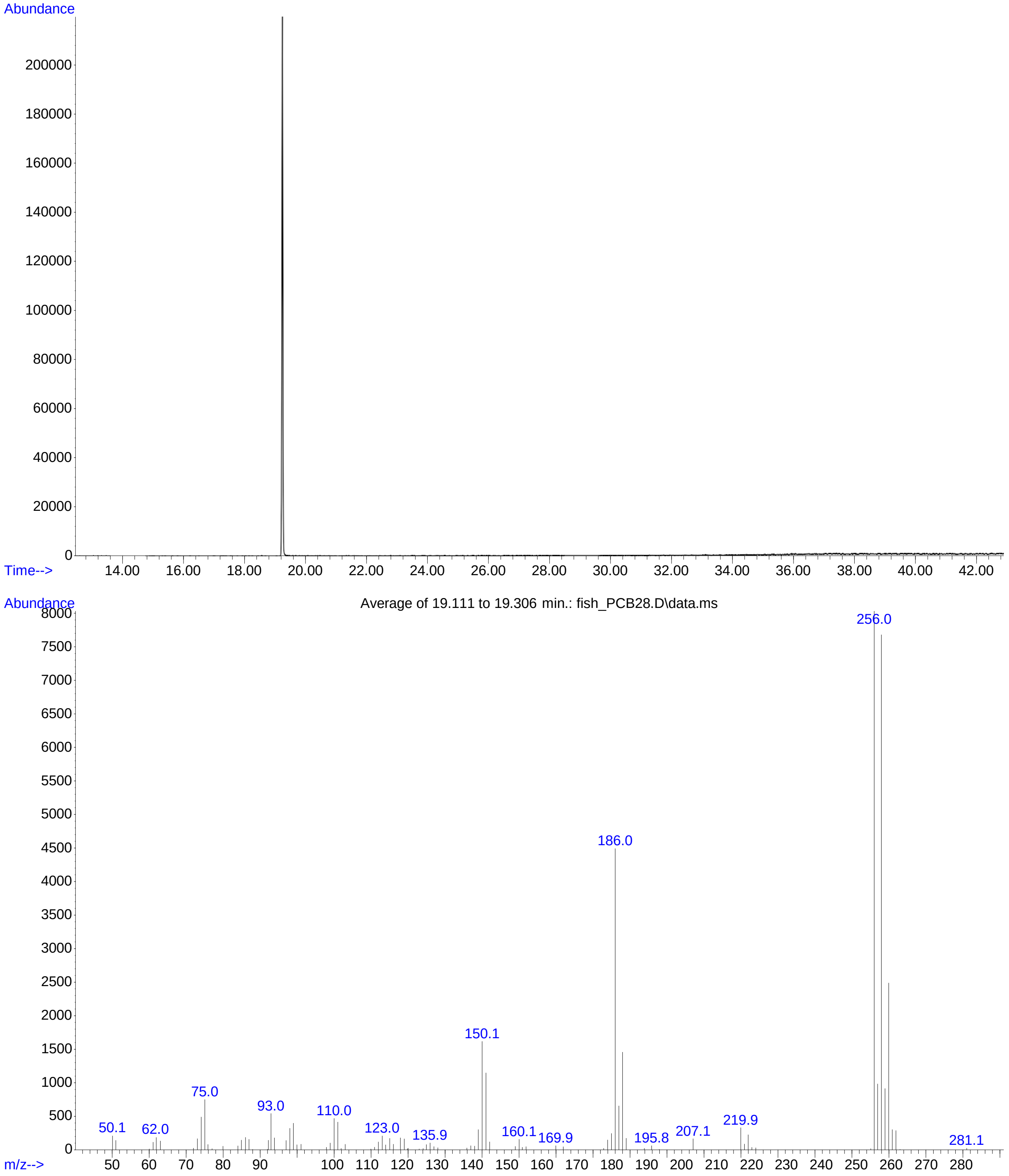

Figure S1 Gas chromatogram and mass spectrum of PCB 28 in TIC mode

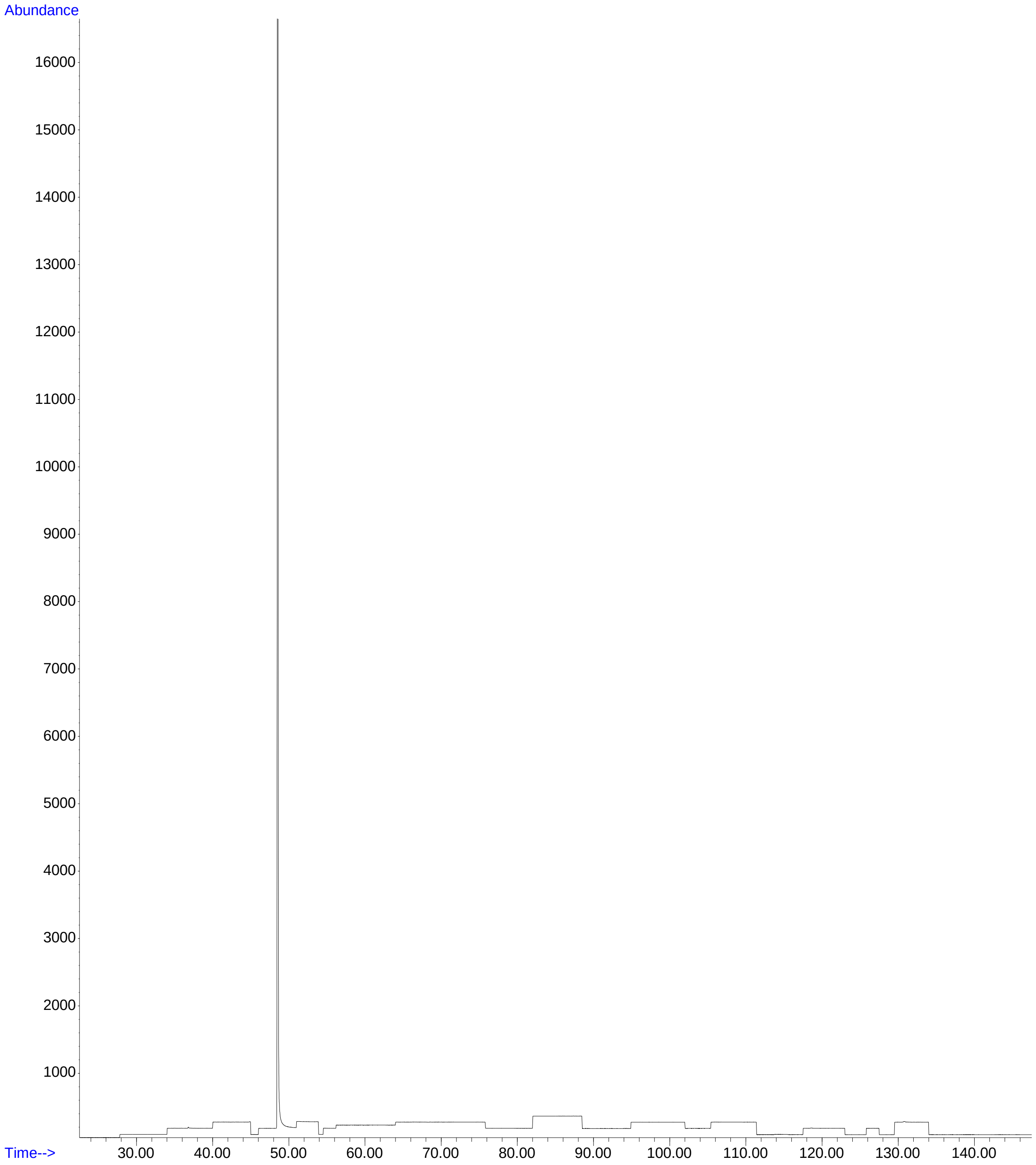

Figure S2 Gas chromatogram of PCB 28 in SIM mode

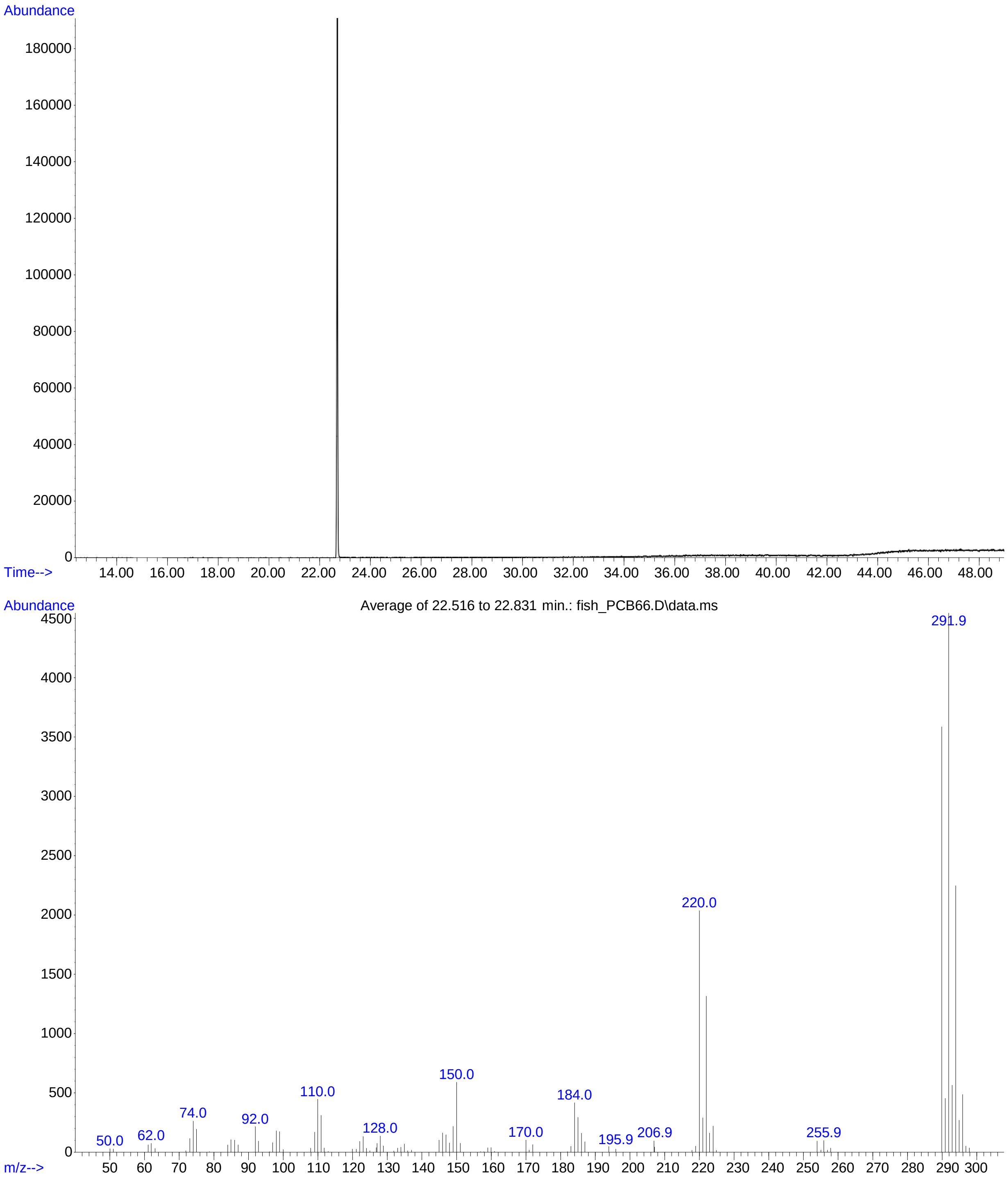

Figure S3 Gas chromatogram and mass spectrum of PCB 66 in TIC mode

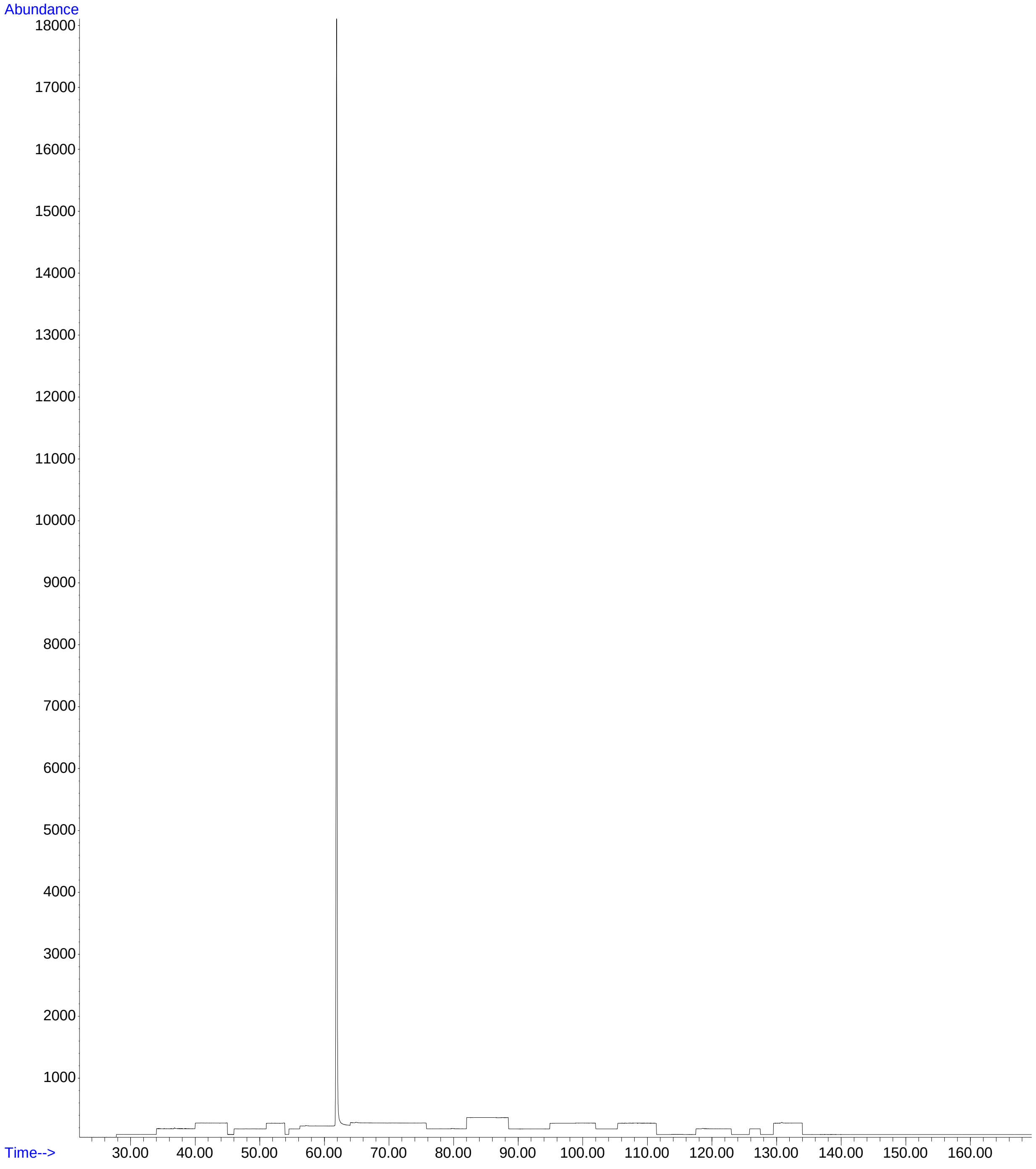

Figure S4 Gas chromatogram of PCB 66 in SIM mode

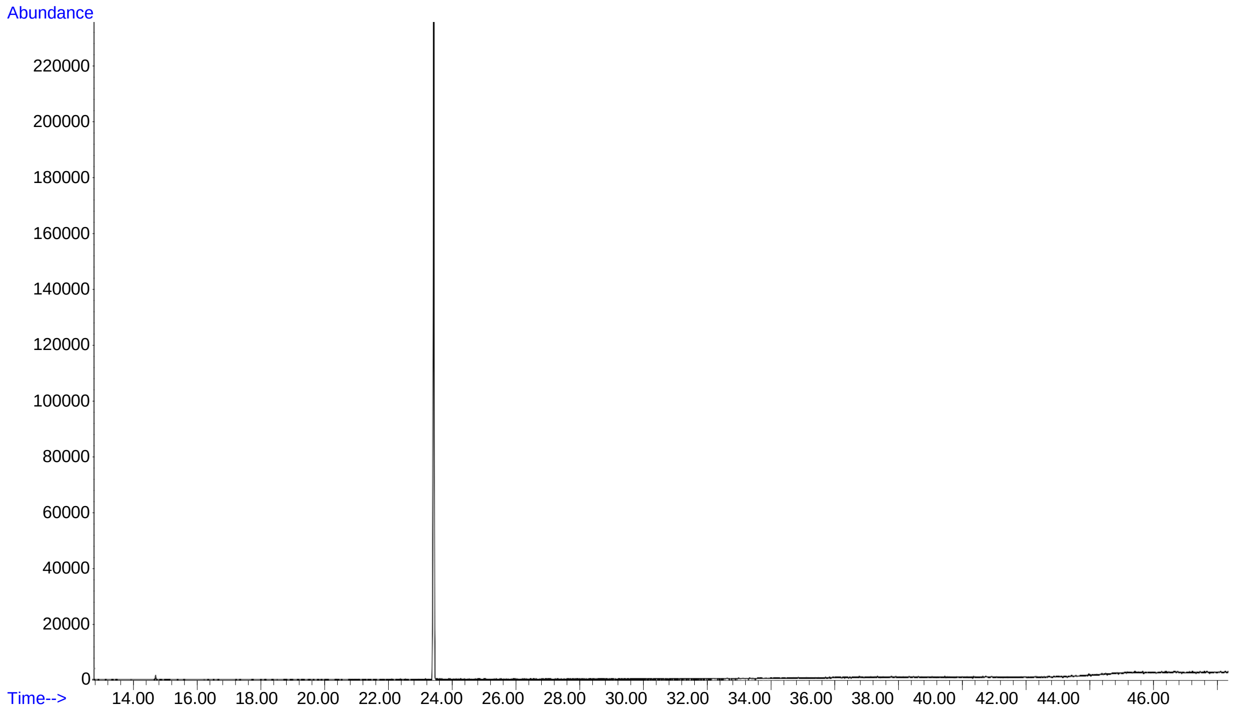

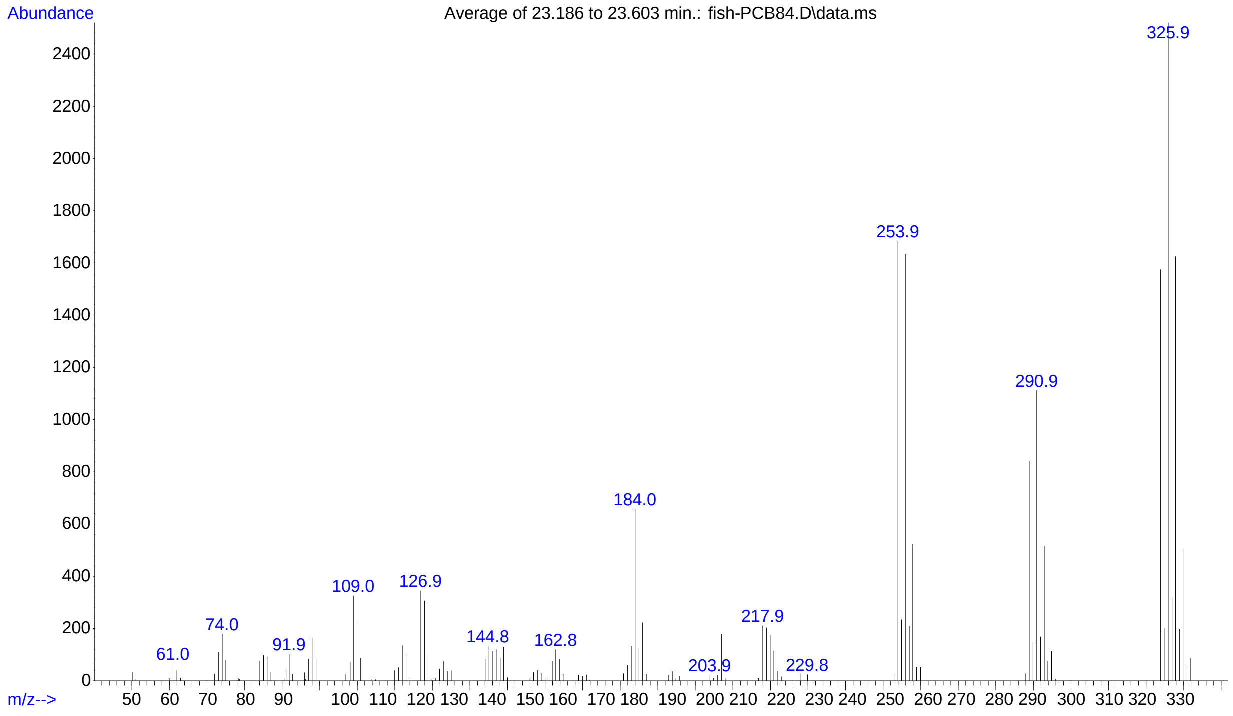

Figure S5 Gas chromatogram and mass spectrum of PCB 84 in TIC mode

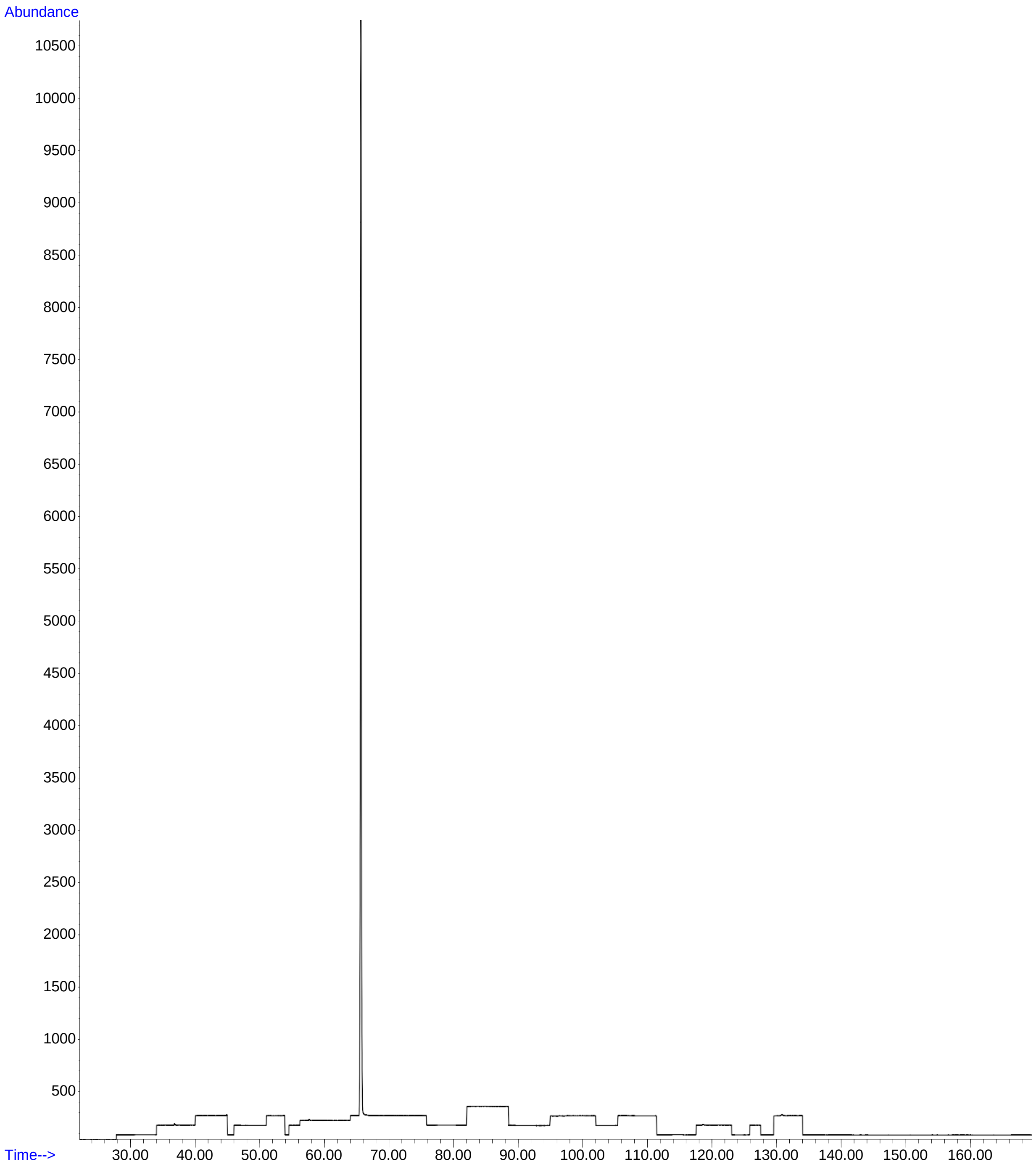

Figure S6 Gas chromatogram of PCB 84 in SIM mode

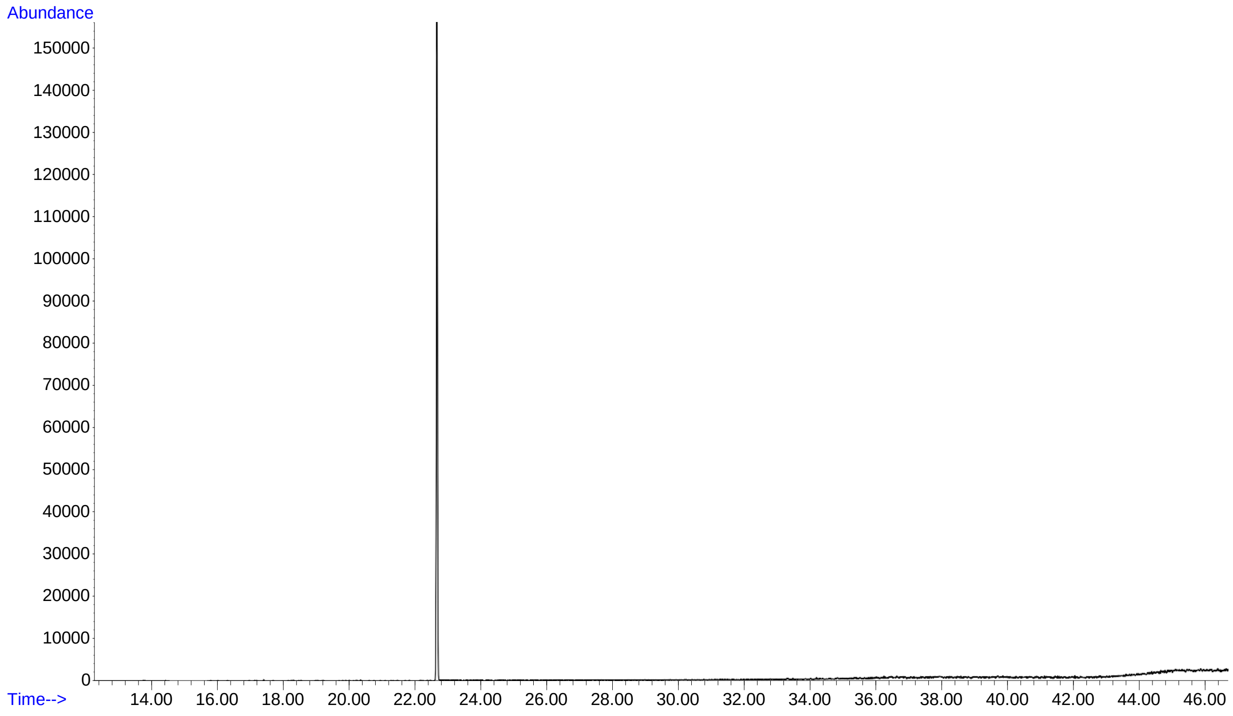

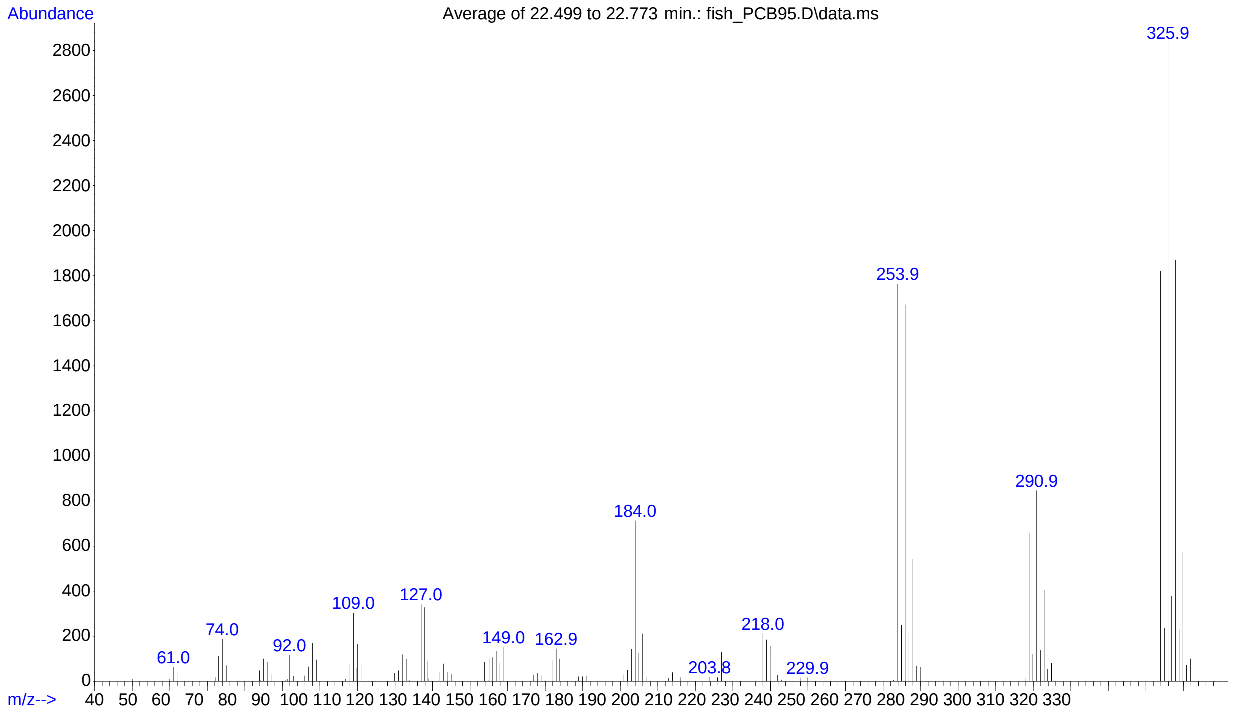

Figure S7 Gas chromatogram and mass spectrum of PCB 95 in TIC mode

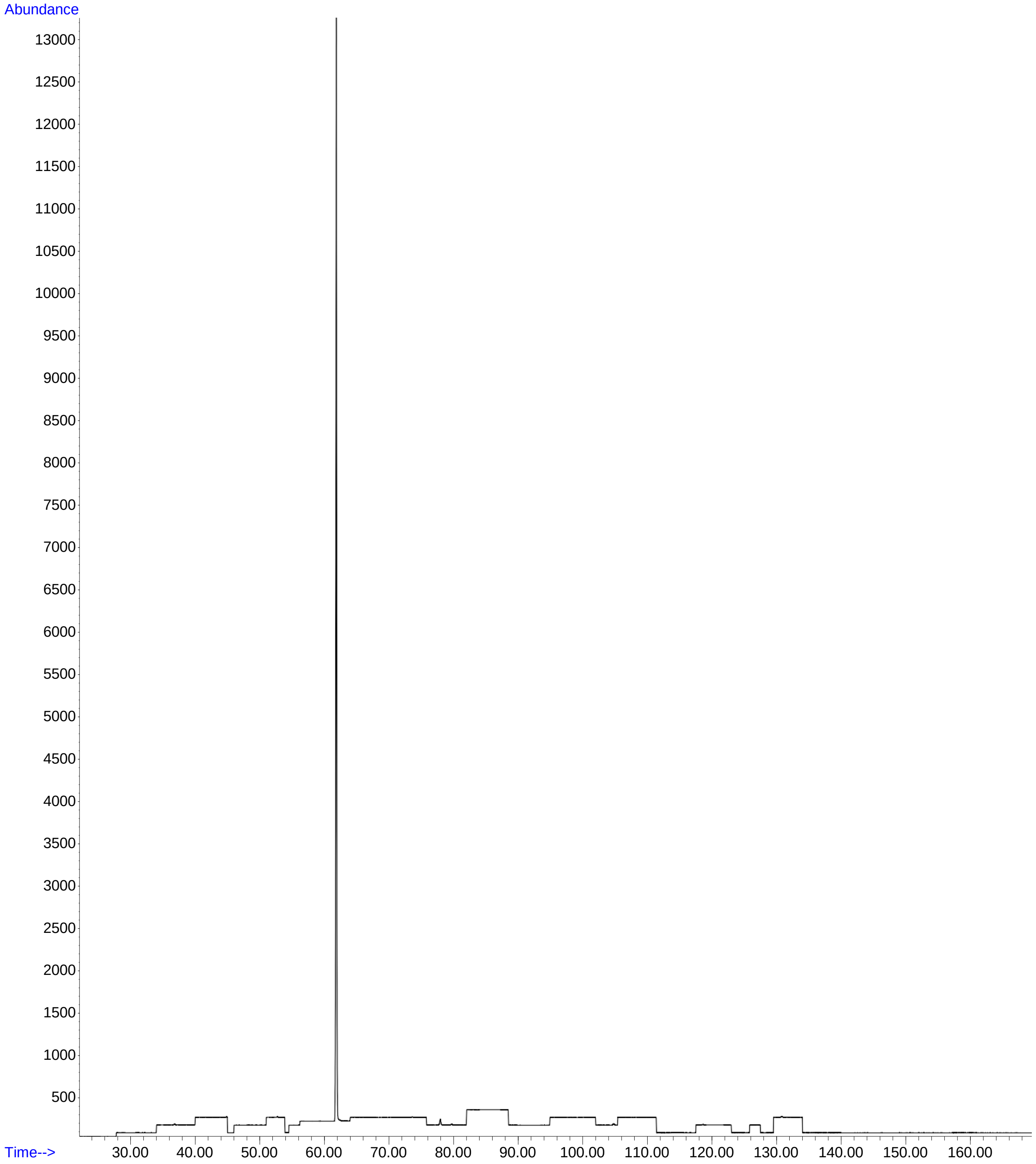

Figure S8 Gas chromatogram of PCB 95 in SIM mode

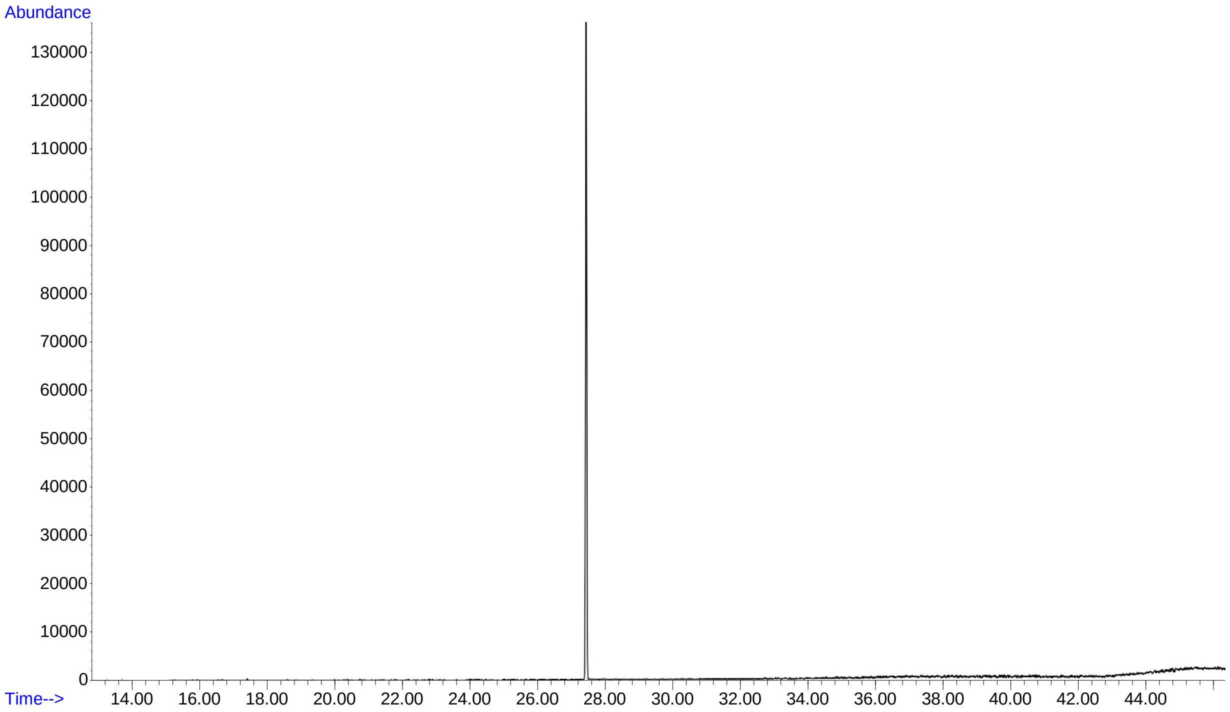

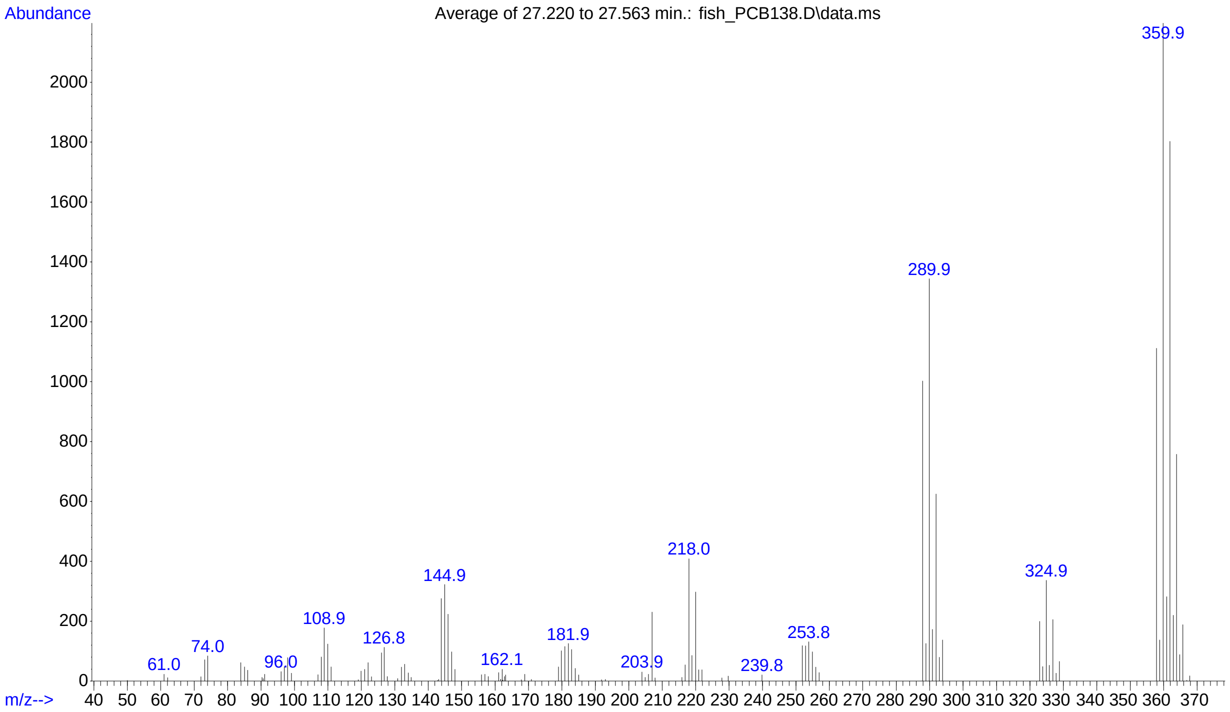

Figure S9 Gas chromatogram and mass spectrum of PCB 138 in TIC mode

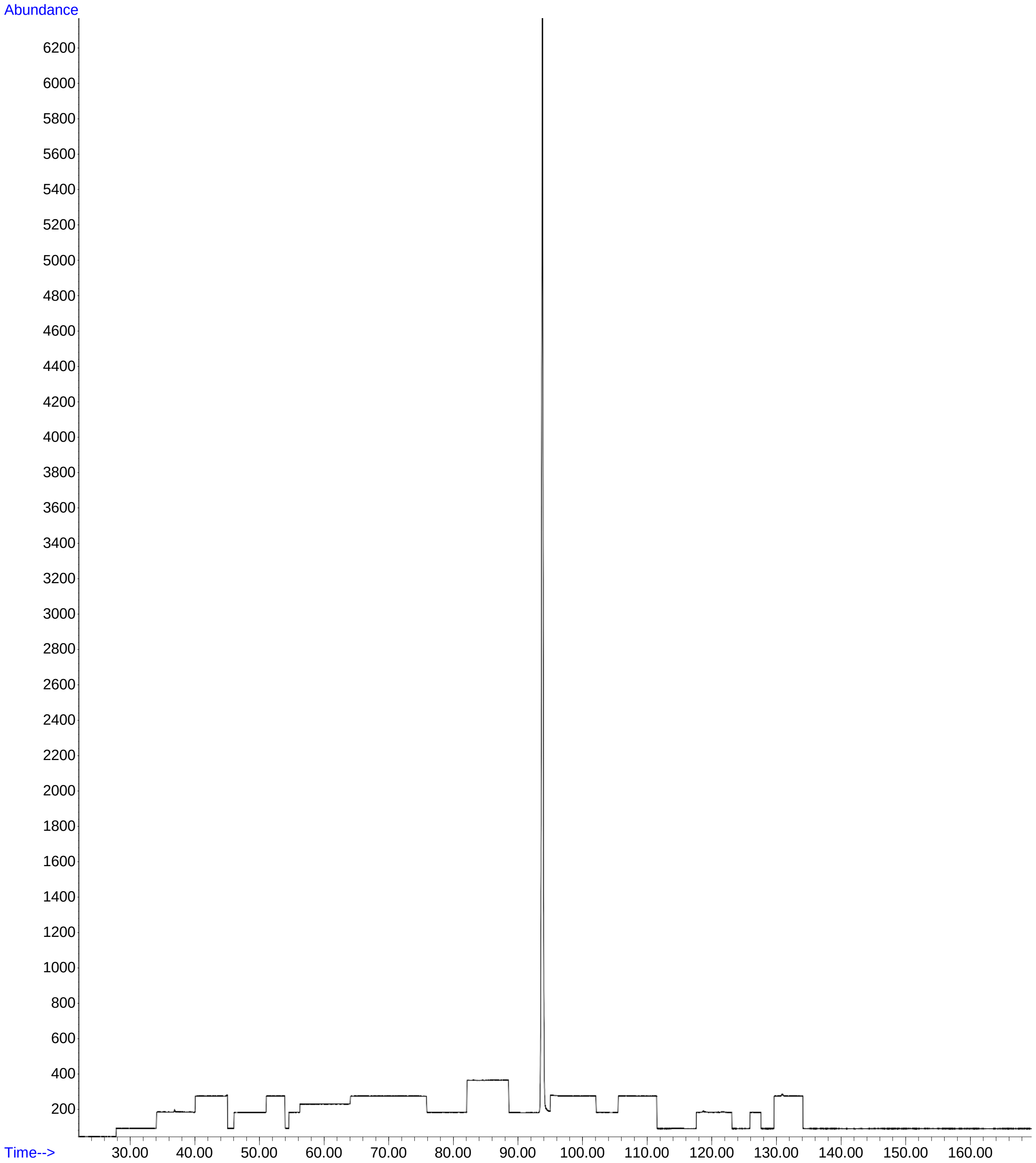

Figure S10 Gas chromatogram of PCB 138 in SIM mode

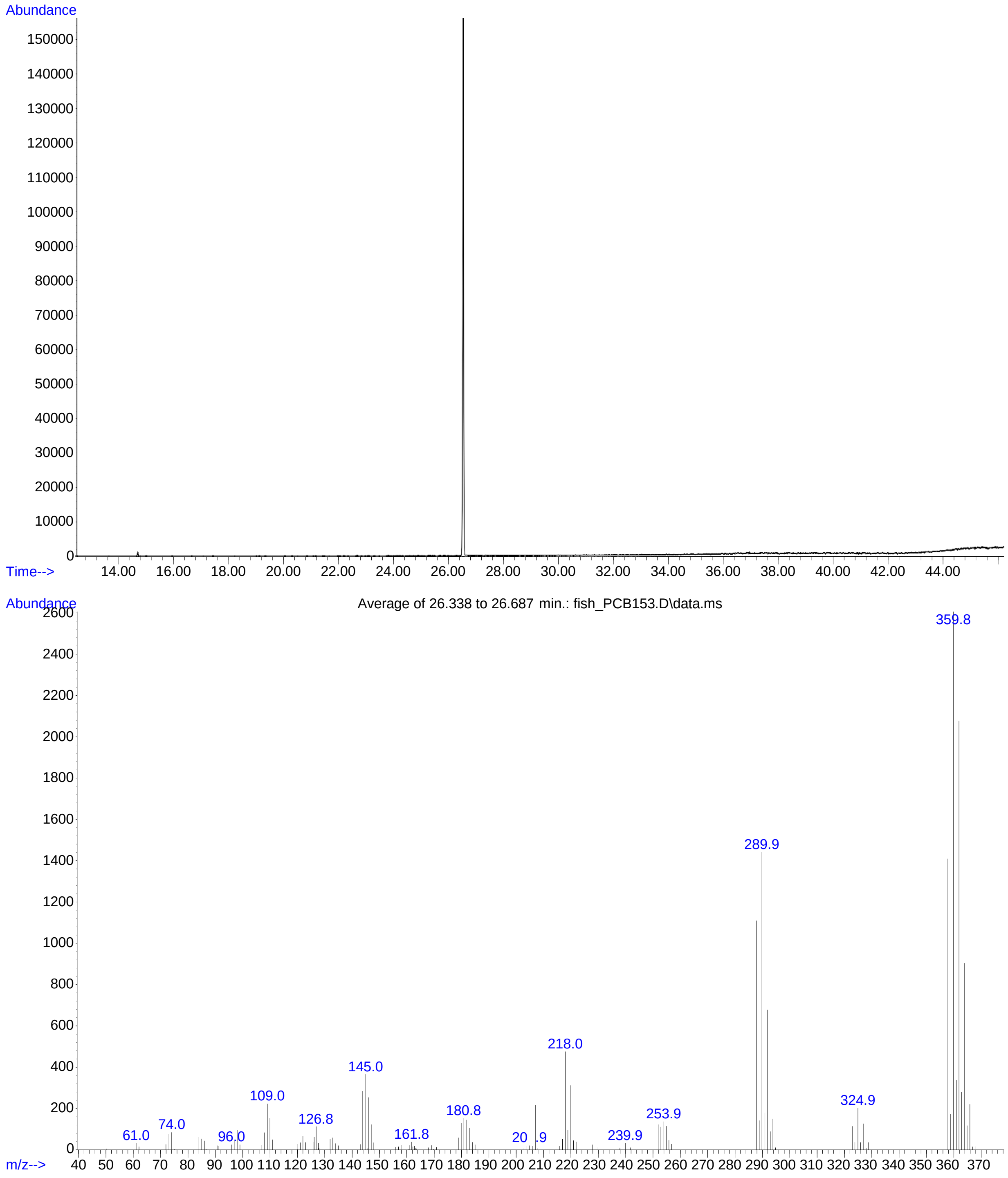

Figure S11 Gas chromatogram and mass spectrum of PCB 153 in TIC mode

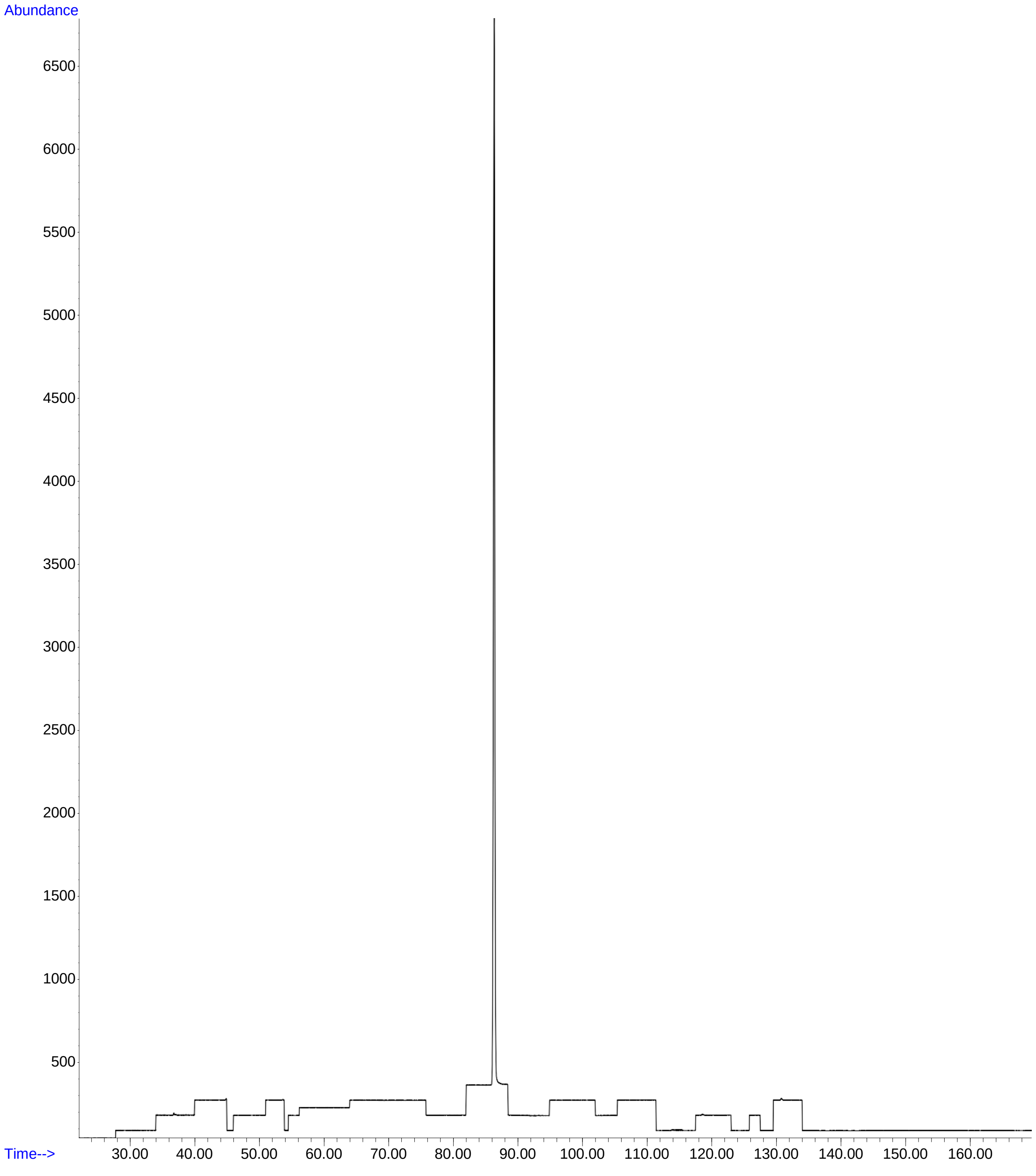

Figure S12 Gas chromatogram of PCB 153 in SIM mode

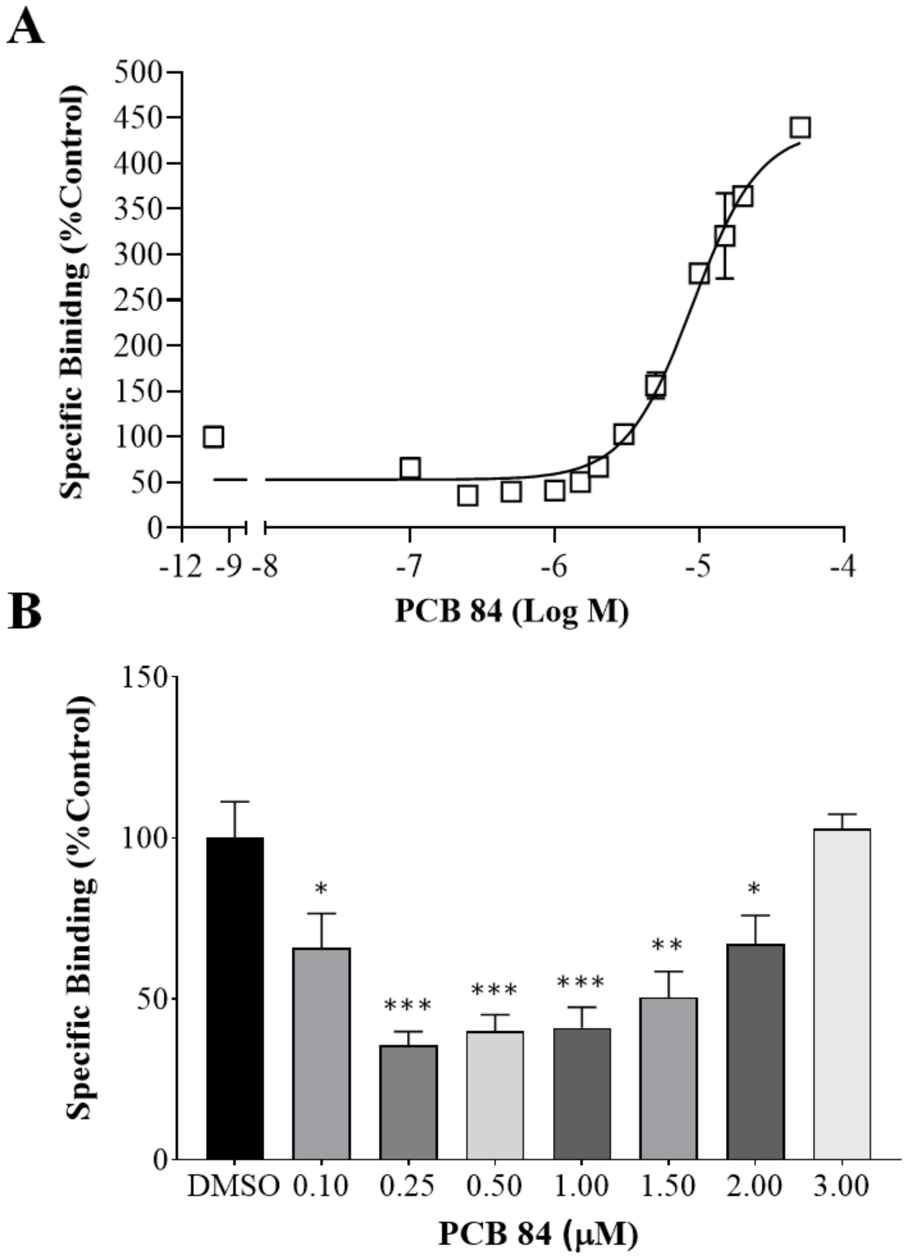

Figure S13 PCB84 displays a non-monotonic response in ryanodine receptor binding assays. A) Full concentration response curve for zebrafish ryanodine receptor exposed to varying PCB 84 concentrations. B) Comparison of lower PCB 84 concentration to the DMSO control using a one-way ANOVA with a Dunn's Post Hoc Analysis. Data represent Means ± SEM for both panel A and B. For B, *p ≤ 0.05, *** p ≤ 0.001, *** p ≤ 0.0001.

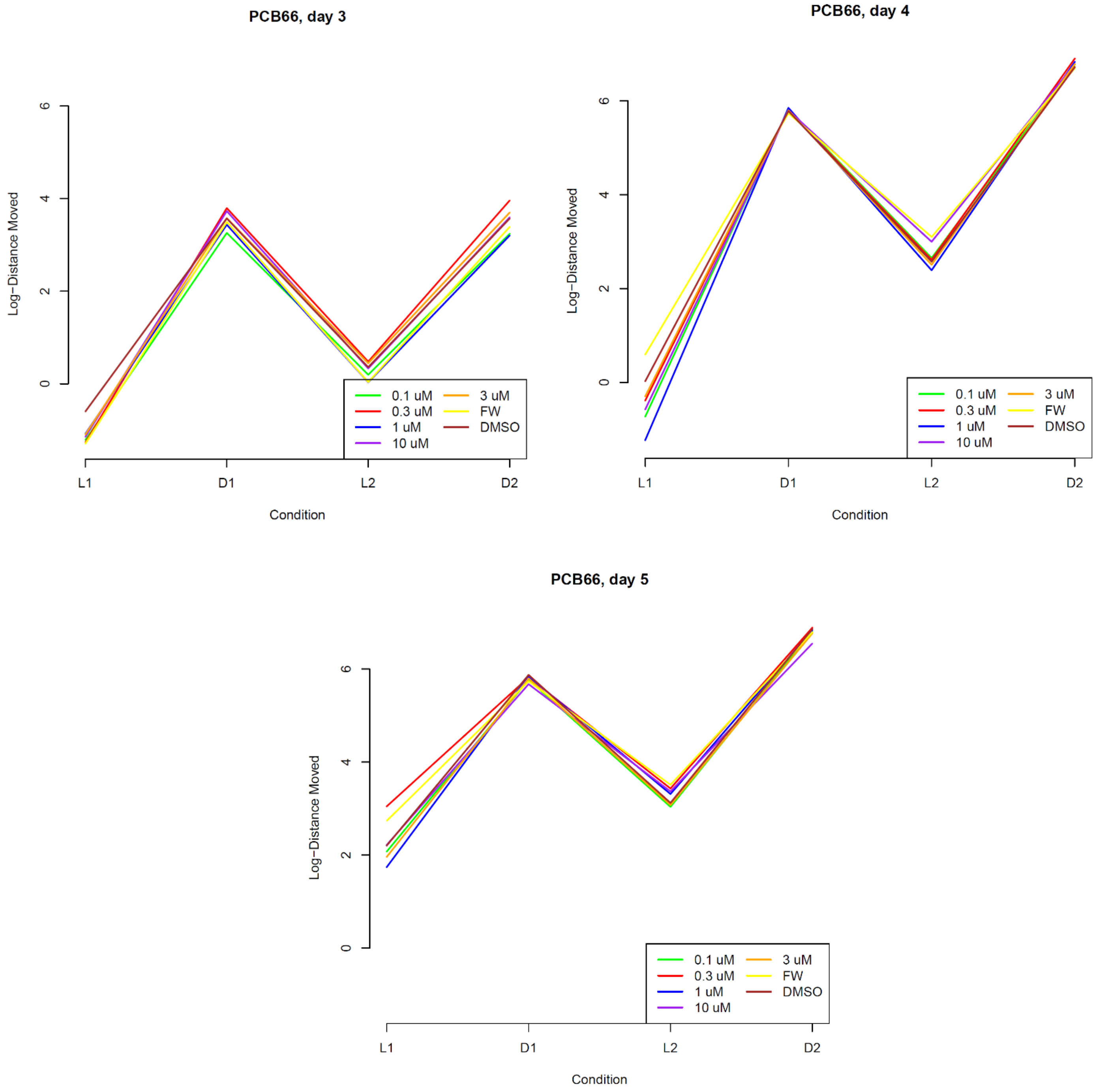

Figure S14 PCB 66 behavioral results. Transitions during light epochs of larvae exposed to PCB 66 (0.1, 0.3, 1, 3, and 10 µM), DMSO control, and FW control at 3, 4, and 5 dpf.

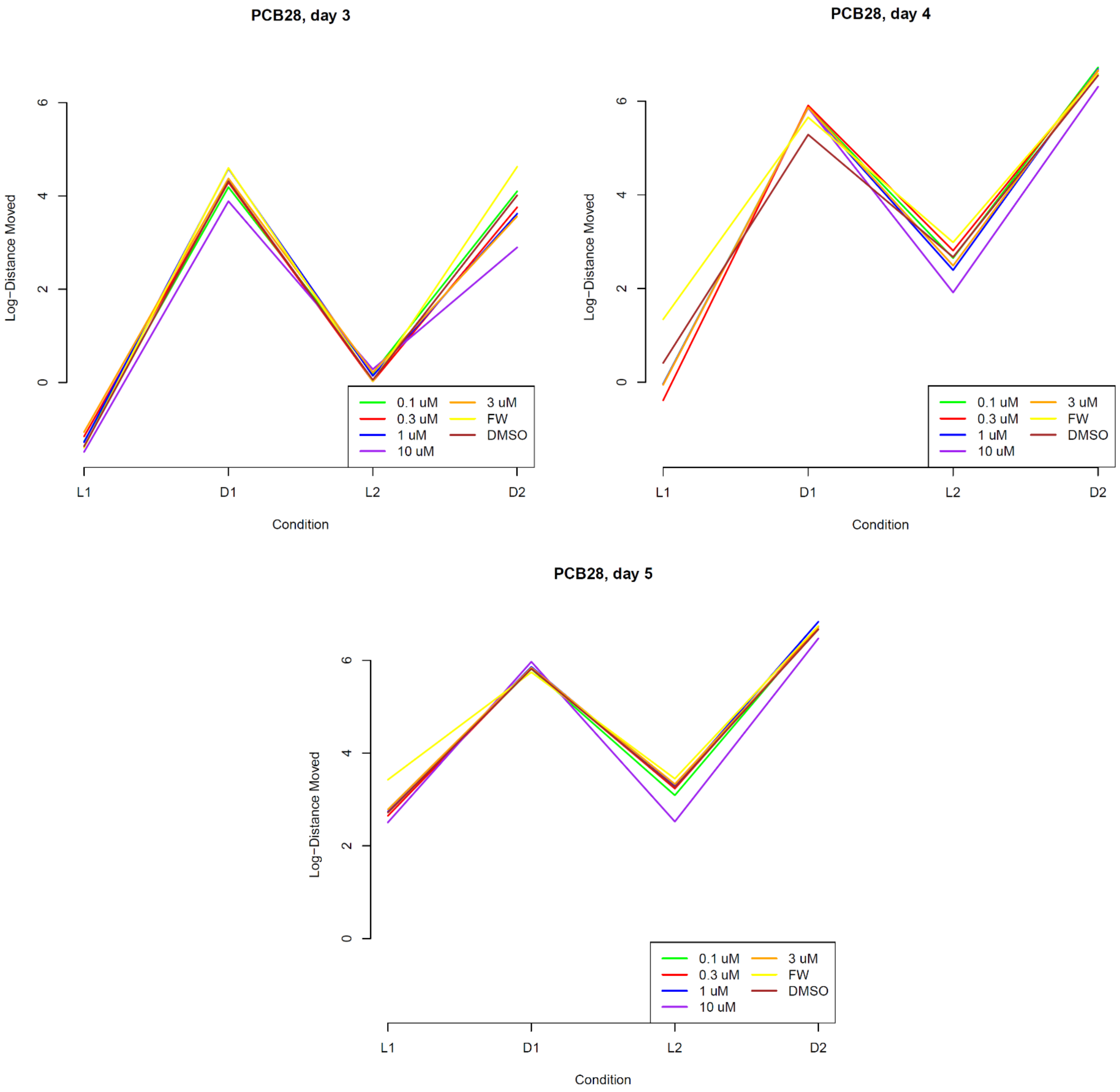

Figure S15 PCB 28 behavioral results. Transitions during light epochs of larvae exposed to PCB 28 (0.1, 0.3, 1, 3, and 10 μM), DMSO control, and FW control at 3, 4, and 5 dpf.

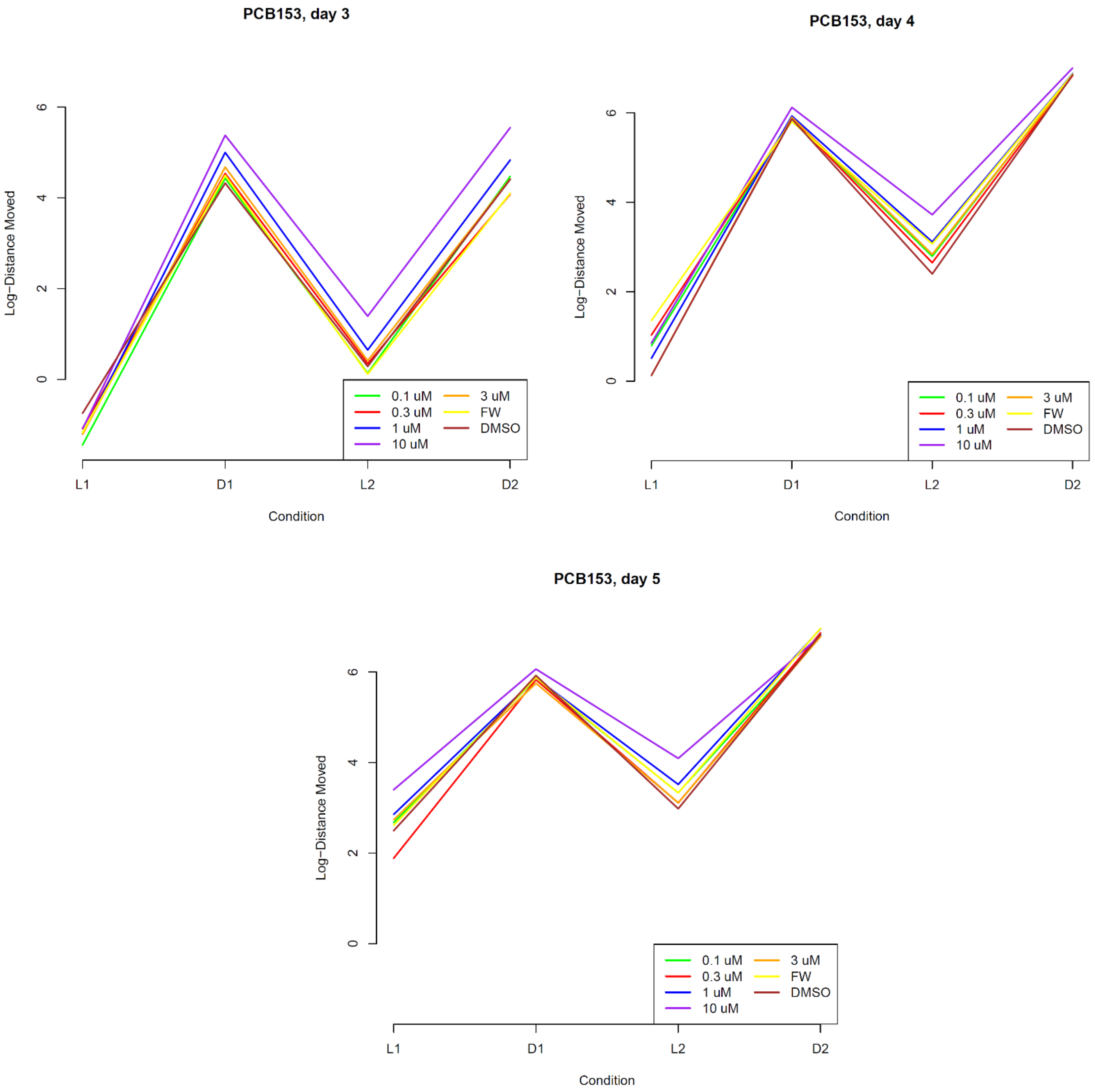

Figure S16 PCB 153 behavioral results. Transitions during light epochs of larvae exposed to PCB 153 (0.1, 0.3, 1, 3, and 10 μM), DMSO control, and FW control at 3, 4, and 5 dpf.

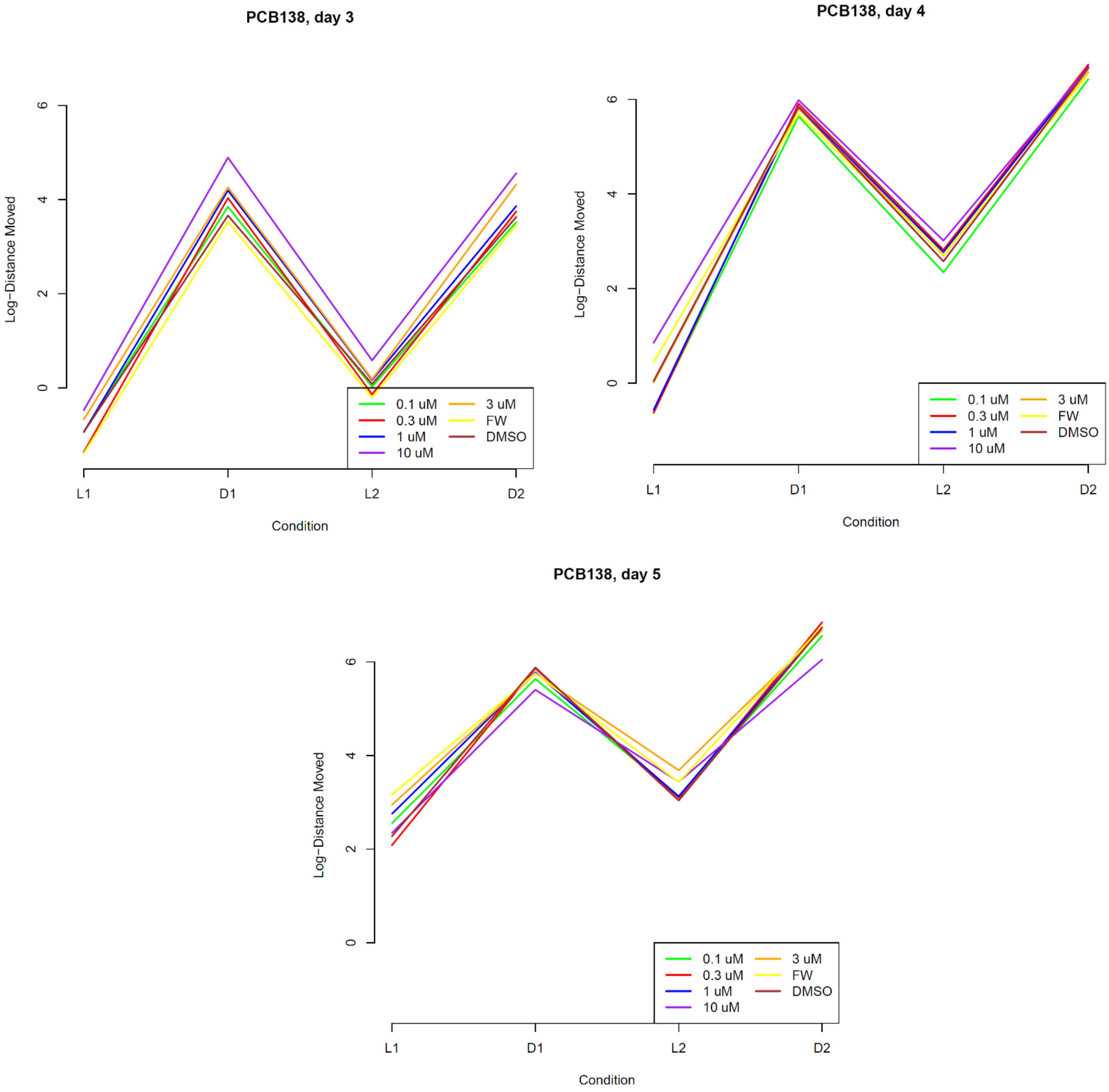

Figure S17 PCB 138 behavioral results. Transitions during light epochs of larvae exposed to PCB 138 (0.1, 0.3, 1, 3, and 10 µM), DMSO control, and FW control at 3, 4, and 5 dpf.

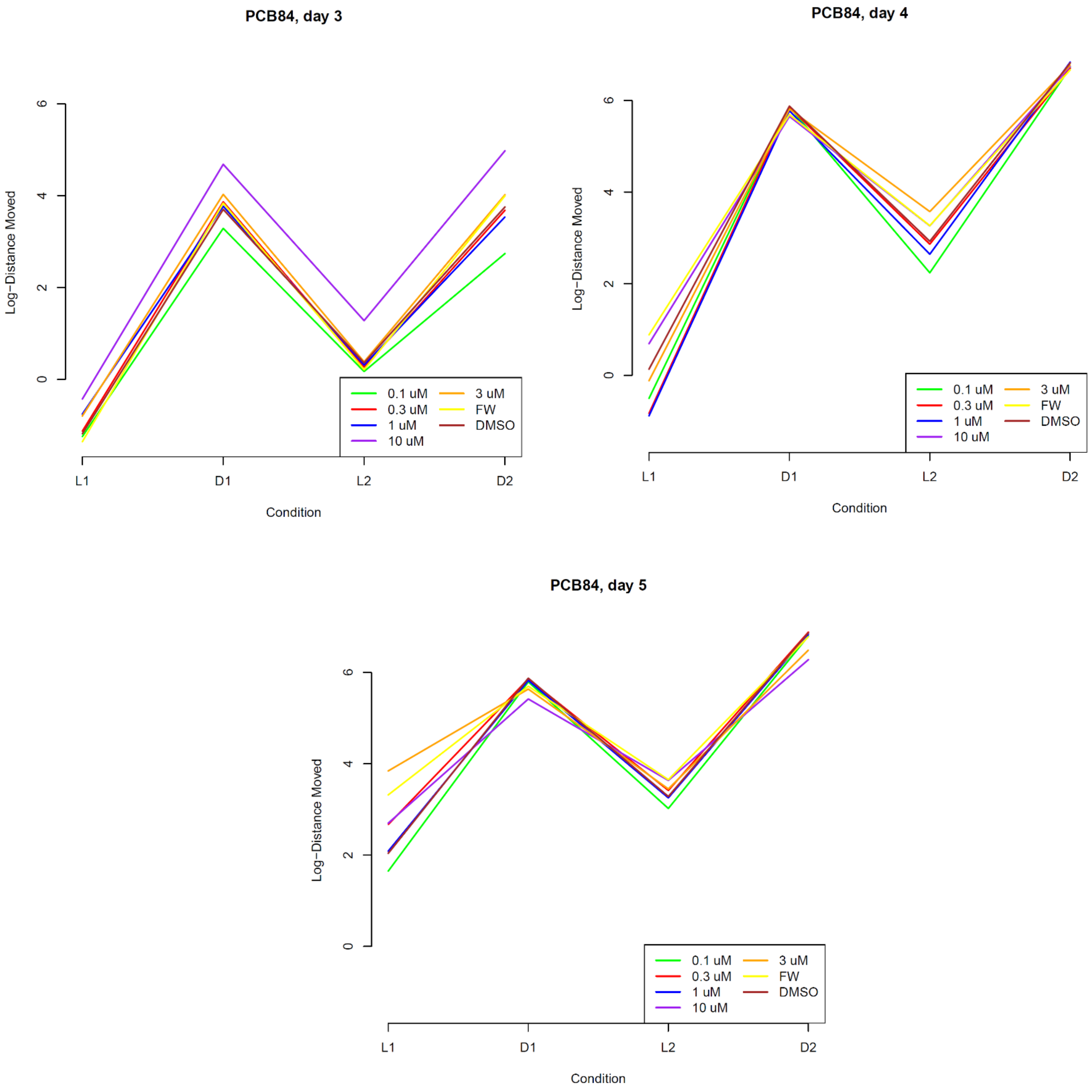

Figure S18 PCB 84 behavioral results. Transitions during light epochs of larvae exposed to PCB 84 (0.1, 0.3, 1, 3, and 10 μM), DMSO control, and FW control at 3, 4, and 5 dpf.

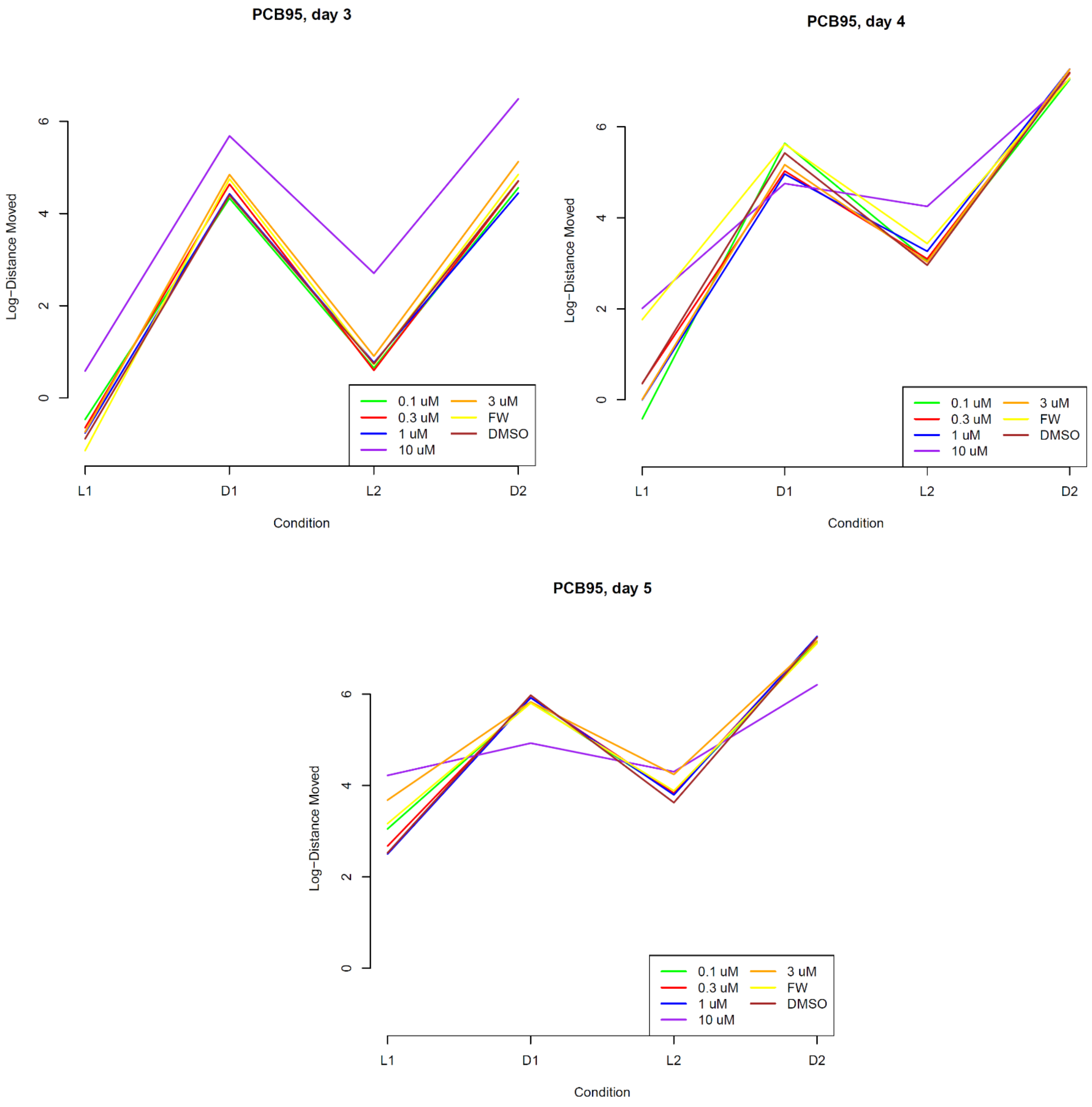

Figure S19PCB 95 behavioral results. Transitions during light epochs of larvae exposed to PCB 95 (0.1, 0.3, 1, 3, and 10 μM), DMSO control, and FW control at 3, 4, and 5 dpf.

Table S1 Limits of detection (*LOD*s), limits of quantification (*LOQ*s), background levels of PCBs in untreated Zebrafish larvae, and percent recovery of method and sample spikes.

QA/QC measure	PCB 66	PCB 28	PCB 153	PCB 138	PCB 84	PCB 95
LOD (ng, n=8)[a]	0.5	1.0	0.8	0.1	0.9	0.8
LOQ (ng, n=8)[b]	5	10	8	1	9	8
Background level (ng/sample, n=7)[c]	0.44 ± 0.22	0.30 ± 0.19	0.25 ± 0.19	0.04 ± 0.04	0.36 ± 0.19	0.31 ± 0.11
Method spike recoveries (%, n=7)[d]	70 ± 8	61 ± 6	87 ± 3	87 ± 4	78 ± 4	78 ± 3
Sample spike recoveries (%, n=3)[e]	80 ± 5	74 ± 1	94 ± 2	94 ± 1	88 ± 2	82 ± 2

[a] The LODs were calculated based on blank samples containing Florisil and diatomaceous earth only. The blank samples were analyzed in parallel with zebrafish samples. The LODs were calculated as $LOD = \bar{x} + k \cdot SD$, where $\bar{x}$ is mean of all blank samples, k is Student's t-value for n-1 degrees of freedom at 99% confidence level, and SD is the standard deviation of the blank measurement.
[b] The LOQ was calculated as LOQ = 10 · LOD. (5)
[c] Background levels were calculated based on both DMSO-treated and untreated zebrafish larvae.
[d] PCB analytes were spiked on the Florisil and diatomaceous earth in parallel with zebrafish larvae samples analyses.
[e] PCB analytes were spiked to untreated and DMSO-treated zebrafish larvae samples analyzed in parallel with PCBs-treated zebrafish samples.

References

1. Kania-Korwel I, Shaikh NS, Hornbuckle KC, Robertson LW, Lehmler H-J. Enantioselective disposition of PCB 136 (2,2',3,3',6,6'-hexachlorobiphenyl) in C57BL/6 mice after oral and intraperitoneal administration. Chirality. 2007;19(1):56-66.
2. Joshi SN, Vyas SM, Duffel MW, Parkin S, Lehmler H-J. Synthesis of sterically hindered polychlorinated biphenyl derivatives. Synthesis. 2011(7):1045-54.
3. Bolgar M, Cunningham J, Cooper R, Kozloski R, Hubballm J. Physical, spectral and chromatographic properties of all 209 individual PCB congeners. Chemosphere. 1995;31(2):2687-705.
4. Nakajoh K, Shibata E, Todoroki T, Ohara A, Nishizawa K, Nakamura T. Measurement of temperature dependence for the vapor pressures of twenty-six polychlorinated biphenyl congeners in commercial Kanechlor mixtures by the Knudsen effusion method. Environmental Toxicology and Chemistry. 2006;25(2):327-36.
5. Kania-Korwel I, Shaikh NS, Hornbuckle KC, Robertson LW, Lehmler HJ. Enantioselective disposition of PCB 136 (2,2 ',3,3 ',6,6 '-hexachlorobiphenyl) in C57BL/6 mice after oral and intraperitoneal administration. Chirality. 2007;19(1):56-66.
